# A Novel Iflavirus Was Discovered in Green Rice Leafhopper *Nephotettix cincticeps* and Its Proliferation Was Inhibited by Infection of Rice Dwarf Virus

**DOI:** 10.3389/fmicb.2020.621141

**Published:** 2021-01-08

**Authors:** Wenxi Jia, Fei Wang, Jingjing Li, Xuefei Chang, Yi Yang, Hongwei Yao, Yanyuan Bao, Qisheng Song, Gongyin Ye

**Affiliations:** ^1^ State Key Laboratory of Rice Biology, Ministry of Agriculture and Rural Affairs Key Laboratory of Molecular Biology of Crop Pathogens and Insects, Institute of Insect Sciences, Zhejiang University, Hangzhou, China; ^2^ Division of Plant Sciences, College of Agriculture, Food and Natural Resources, University of Missouri, Columbia, MO, United States

**Keywords:** Iflaviridae, Nephotettix cincticeps, transmission, covert infection, rice dwarf virus, distribution, co-infection, virus-virus interaction

## Abstract

The green rice leafhopper, *Nephotettix cincticeps* (Hemiptera: Cicadellidae), is a key insect vector transmitting rice dwarf virus (RDV) that causes rice dwarf disease. We discovered a novel iflavirus from the transcriptomes of *N. cincticeps* and named it as Nephotettix cincticeps positive-stranded RNA virus-1 (NcPSRV-1). The viral genome consists of 10,524 nucleotides excluding the poly(A) tail and contains one predicted open reading frame encoding a polyprotein of 3,192 amino acids, flanked by 5' and 3' untranslated regions. NcPSRV-1 has a typical iflavirus genome arrangement and is clustered with the members of the family *Iflaviridae* in the phylogenetic analysis. NcPSRV-1 was detected in all tested tissues and life stages of *N. cincticeps* and could be transmitted horizontally and vertically. Moreover, NcPSRV-1 had high prevalence in the laboratory populations and was widely spread in field populations of *N. cincticeps*. NcPSRV-1 could also infect the two-striped leafhopper, *Nephotettix apicalis*, at a 3.33% infection rate, but was absent in the zigzag leafhopper, *Recilia dorsalis*, and rice *Oryza sativa* variety TN1. The infection of RDV altered the viral load and infection rate of NcPSRV-1 in *N. cincticeps*, for which it seems that RDV has an antagonistic effect on NcPSRV-1 infection in the host.

## Introduction

It has long been acknowledged that insects are important vectors and hosts of numerous viruses infecting humans, animals, and plants. Many viruses are found because of the symptomatic diseases they caused (Nouri et al., 2018). For example, the iflaviruses are initially isolated from several economic insects, i.e., honey bees and silkworms, causing flacherie, malformations, and death of the infected insects (Isawa et al., 1998; Ghosh et al., 1999). However, over the past decade, with the development of next-generation sequencing and bioinformatics tools, numerous viruses producing covert infections have been discovered from the asymptomatic hosts.

*Iflaviridae* is a family classified under the order *Picornavirales*, comprising viruses with monopartite, single-stranded, positive-sense, and non-segmented RNA genomes. Currently, the family *Iflaviridae* consists of one genus *Iflavirus*, with 15 classified members. To date, all the reported iflaviruses infect exclusively arthropod hosts, especially insects. The insect hosts of iflaviruses have been found in the orders including Hemiptera, Lepidoptera, Hymenoptera, Coleoptera, and Diptera (van Oers, 2010; Valles et al., 2017). Up to February, 2020, 12 iflavirus species have been reported within Hemiptera according to the PubMed database and the species demarcation criteria of *Iflaviridae* on ICTV, including planthopper iflaviruses such as Nilaparvata lugens honeydew virus-1 (NlHV-1), Nilaparvata lugens honeydew virus-2 (NlHV-2), Nilaparvata lugens honeydew virus-3 (NlHV-3; Murakami et al., 2013, 2014), Laodelphax striatella iflavirus 1 (LsIV1), Laodelphax striatella honeydew virus 1 (LsHV1; Wu et al., 2019), Sogatella furcifera honeydew virus 1 (SFHV1; Wu et al., 2018) and Lygus lineolaris virus 1 (LyLV-1; Perera et al., 2012), aphid iflavirus Brevicoryne brassicae virus (BrBV; Ryabov, 2007), stink bug iflavirus Halyomorpha halys virus (HhV; Sparks et al., 2013), and also leafhopper iflaviruses such as Euscelidius variegates virus 1 (EVV-1; Abba et al., 2017), Psammotettix alienus iflavirus 1 (PaIV1; Li et al., 2019), and Graminella nigrifrons virus 1 (GNV-1; Chen et al., 2015). Iflaviruses have also been found in bats feces, showing high sequence identities to insect iflaviruses (Yinda et al., 2017; Simic et al., 2020). For example, bat iflavirus is closely related to slow bee paralysis virus and the nucleotide composition analysis of the viral genome indicates that the iflavirus may have insect hosts (Yinda et al., 2017). Thus, it suggests that the iflaviruses found in bats feces might be diet derived from the infected insects (Yinda et al., 2017).

The virions of *Iflaviridae* are non-enveloped, roughly spherical particles with a diameter of 22–30 nm (Wang et al., 1999; Ongus et al., 2004; Valles et al., 2017). Capsid proteins VP1, VP2, and VP3 [viral protein (VP)] generated from a polyprotein form one protomer. Virions are made of 60 protomers and exhibit pseudo-T3 icosahedral symmetry. Iflaviruses are supposed to harbor short VP4 subunits which could facilitate the genome delivery of many picornaviruses and dicistroviruses; however, the VP4 subunits have not been detected and established in iflaviruses yet (Kalynych et al., 2016; Skubnik et al., 2017; Prochazkova et al., 2018). The replication and accumulation of iflavirus occur in the cytoplasm and are also in association with membranes (Zhang et al., 2007; van Oers, 2010; Valles et al., 2017). The virion RNA, containing approximately 9–11 kilobases in length, serves as both the genome and mRNA and contains a single open reading frame (ORF) encoding one large polyprotein (van Oers, 2010; Valles et al., 2017). The ORF is flanked by 5' and 3' untranslated regions (UTRs) that contain functional secondary RNA structures, such as internal ribosome entry site (IRES), mediating the viral translation and replication, and the size of the UTRs varies between the species (Lu et al., 2006; Ongus et al., 2006; Wu et al., 2007; Li et al., 2012). Besides, a genome-linked virus protein (VPg) is covalently attached to the 5' terminus of the viral genome (Hashimoto et al., 1986; van Oers, 2010; Valles et al., 2017). And also, the presence of a leader protein has been reported in several iflaviruses, such as deformed wing virus (DWV), varroa destructor virus-1 (VDV-1), slow bee paralysis virus (SBPV), and infectious flacherie virus (IFV) (Isawa et al., 1998; Lanzi et al., 2006; de Miranda et al., 2010; de Miranda and Genersch, 2010). The polyprotein is processed co- and post-translation by proteolytic cleavage (*via* viral proteases) into functional viral proteins (van Oers, 2010; Jiang et al., 2014). The typical iflavirus genome arrangement is represented as follows: VPg + 5'UTR^IRES^[L-VP2-Vp4-VP3-VP1/-Helicase(2C^ATPase^)-protease(3C^PRO^)-RdRp(3D^Pol^)]3'UTR-poly(A) (van Oers, 2010; Valles et al., 2017).

The green rice leafhopper (GRLH), *Nephotettix cincticeps* (Hemiptera: Cicadellidae), is one of the important pests on rice in Asia and could lead to the loss of rice yields (Ruan et al., 1981; Cheng, 1996). *N. cincticeps* could damage rice plants *via* direct ingestion and secondary virus transmission, such as rice dwarf virus (RDV). RDV, a double-stranded phytoreovirus of the family *Reoviridae*, is the pathogen of rice dwarf disease and mainly transmitted by *N. cincticeps* in a persistent-propagative manner (Hibino, 1996; Honda et al., 2007; Hogenhout et al., 2008; Wei and Li, 2016). RDV influences both rice and *N. cincticeps* by a complex host-virus-vector interaction (Wang et al., 2018; Wei et al., 2018). It is of great importance to obstruct or suppress the viral replication in the insect vector, thereby cutting off the transmission route of the virus.

In the present study, we assembled and characterized the genome sequence of the picorna-like virus discovered from the transcriptomes of RDV-infected and uninfected *N. cincticeps* and named it as Nephotettix cincticeps positive-stranded RNA virus-1 (NcPSRV-1). NcPSRV-1 shares a typical iflavirus genome organization. The sequence alignments and phylogenic analysis suggest that NcPSRV-1 belongs to the family *Iflaviridae*. Our findings reveal the characteristics of the viral prevalence, transmission, and co-infection with RDV of NcPSRV-1.

## Materials and Methods

### Insect Populations

Five laboratory populations of the green rice leafhopper *N. cincticeps*, and two laboratory populations of the zigzag leafhopper *Recilia dorsalis* (Hemiptera: Cicadellidae) and the two-striped leafhopper *Nephotettix apicalis* (Hemiptera: Cicadellidae) were used in this study. For *N. cincticeps*, population A was originally collected from rice fields on the Zijingang Campus of Zhejiang University, Hangzhou, China in October, 2014 and was reared on healthy rice plants (*Oryza sativa* variety TN1). Population B was originally collected from rice fields on zhe Zijingang Campus of Zhejiang University, Hangzhou, China in November, 2015 and was reared on healthy TN1 rice plants. Population C was originally collected together with population B at the same time and fields and was reared on RDV-infected TN1 rice plants. Population D was originally collected together with population A at the same time and fields and was reared on RDV-infected TN1 rice plants. The RDV infection rates of *N. cincticeps* in population D were more than 90%. NcPSRV-1 negative population was originally collected from rice fields in Deqing, Zhejiang, China, in July, 2020 and was reared on healthy TN1 rice plants after NcPSRV-1 detection.

For *R. dorsalis*, the population was originally collected from rice fields on the Zijingang Campus of Zhejiang University, Hangzhou, China in 2016. The population of *N. apicalis* was originally collected from rice fields in Xundian Hui and Li autonomous county, Yunnan Province, China in 2019.

All laboratory populations were maintained within insect-proof cages in the climate chamber (Wang et al., 2017) and kept at 26 ± 1°C, 60 ± 5% relatively humidity with a 16:8 h (L/D) photoperiod.

### RNA Extraction and Virus Genome Sequencing

Total RNA was extracted from the 5th instar nymphs of *N. cincticeps* using TRIzol (Invitrogen, California, United States). RNA concentration of each sample was determined using a Nanodrop 2000 spectrophotometer (Thermo Scientific, Wilmington, DE, United States). Reverse transcription reactions (1 μg total RNA per sample) was performed using the TransScript One-Step gDNA Removal and cDNA Synthesis SuperMix Kit (TransGen Biotech, Beijing, China) according to the manufacturer’s instructions and the synthesized cDNA was amplified as a template for polymerase chain reaction (PCR).

Salivary glands of the 5th instar nymphs from population A and D were collected for RDV-uninfected and infected transcriptome analyses with three biological replicates, respectively (Novogene, Beijing, China). Based on the virus genome-like sequences identified in RNA-seq assembled sequences, 15 pairs of primers (Supplementary Table S1) were designed to generate overlapping PCR fragments covering the full-length genome in order to verify the virus genome sequence. PCR products were purified and sequenced.

The 5' and 3' ends of the viral genome were confirmed by 5' and 3' rapid amplification of cDNA ends (RACE) according to the instructions of SMART RACE cDNA amplification Kit (Clontech, CA, United States). The amplified 5' and 3' RACE products were cloned into pRACE vector and sequenced.

### Bioinformatic and Phylogenetic Analysis

Nucleotide sequence analysis and assembly of virus genome were performed *via* Vector NTI (Li and Moriyama, 2004). The ORF of NcPSRV-1 genome was predicted using ORF finder on NCBI^1^ and Vector NTI. The secondary structure predictions of 5' UTR and 3' UTR were assessed on RNAfold^2^ and Mfold.^3^

Conserved domains of the deduced amino acid (aa) sequence were predicted using Protein BLAST on NCBI^4^ and InterPro.^5^ The genome sequences of representative species used for multiple sequence alignments were selected according to the taxonomy of order *Picornavirales* on ViralZone^6^ and ICTV,^7^ and also the Protein BLAST results. Sequences of deduced RNA-dependent RNA polymerase (RdRP) domain were aligned through MUSCLE in MEGA7 (Kumar et al., 2016). The best-fit substitution model for phylogenic reconstruction was predicted by IQ-TREE (Nguyen et al., 2015). Using Blosum62 + I + G4 as the best identified substitution model, the phylogenetic reconstruction was conducted by IQ-TREE with a maximum likelihood tree method of 1,000 bootstraps. The phylogenic tree was visualized and edited by iTOL.^8^ Virus abbreviations and accession numbers are shown in Supplementary Table S2.

### Virus Detection and Quantification

RNA extraction and cDNA synthesis were performed as described above. Primers NcPSRV-6F/NcPSRV-6R and NcPSRV-15F/NcPSRV-15R (Supplementary Table S1) were used to detect NcPSRV-1 presence and amplify 897 base pair (bp) and 507 bp PCR products, respectively. PCR program used was as follows: 10 s at 98°C, 10 s at 51°C/56°C, and 5 s at 68°C for 35 cycles. Amplifications were visualized by 1% agarose gel electrophoresis and GelRed staining.

Quantitative real-time polymerase chain reaction (qRT-PCR) was performed to quantify the viral loads of NcPSRV-1 in different *N. cincticeps* samples using virus-specific primers NcPSRV-1S/NcPSRV-1A (Supplementary Table S1), which was located in the VP2 domain. *Beta-actin* of *N. cincticeps* was amplified using primers NcActin-1S/NcActin-1A (Supplementary Table S1) as the reference gene. Amplifications were carried out in 20 μl reaction volume, containing 10 μl SYBR Premix Ex Taq II (Tli RNaseH Plus; Takara Bio, Otsu, Japan), 2 μl primers (10 μM), 2 μl diluted sample cDNA (diluted 1:100), and 6 μl RNase-free water. qRT-PCR was conducted using the Bio-Rad CFX 96 Real-Time Detection System (Bio-Rad, Hercules, CA, United States). The thermal cycling program was: 94°C for 30 s, 40 cycles of 95°C for 5 s, and 55°C for 30 s, followed by a final melting curve. The Ct values of NcPSRV-1 were normalized by *beta-actin* using the comparative 2^-ΔΔCt^ method (Livak and Schmittgen, 2001).

### Tissue Distribution and Developmental Expression Patterns of NcPSRV-1

To detect the spatial distribution pattern of NcPSRV-1 in *N. cincticeps*, brains, salivary glands, midguts, fat bodies, and carcasses were dissected from the 5th instar nymphs, ovaries from females, and testes from male adults. The insects collected from population A were anesthetized on ice before dissection. The dissection was performed in phosphate buffered saline (PBS, 0.01 M, pH 7.4) using a pair of forceps under dissecting microscope (Leica, Wetzlar, Germany). The tissues were instantly moved into TRIzol and 50 tissues of one kind were pooled as one biological replicate. Three biological replicates were performed. The viral loads of NcPSRV-1 in different tissues were quantified using qRT-PCR.

To detect the developmental expression pattern at different stages of NcPSRV-1 in *N. cincticeps*, eggs, the 1st-5th instar nymphs, female and male adults were collected from population A and transferred into TRIzol. The eggs were collected from different egg masses. Fifty eggs/nymphs or adults (per stage/sex) were pooled as one biological replicate and each life stage was performed in triplicate. The total RNA of different life stages was extracted. The cDNA synthesis and qRT-PCR of different samples were conducted as described above.

### Detection of NcPSRV-1 in Field Populations of *N. cincticeps*

To investigate the prevalence of NcPSRV-1 in *N. cincticeps* field populations, *N. cincticeps* populations were collected from different locations in China in 2017, including Hangzhou (119.94°E, 30.05°N), Jinhua (119.64°E, 29.08°N), Wuhan (113.96°E, 30.47°N), Hefei (116.78°E, 31.72°N), Guiyang (106.56°E, 26.50°N), Fuzhou (119.39°E, 26.10°N), Changsha (113.17°E, 28.18°N), Kaifeng (114.45°E, 34.80°N), Guangzhou (113.35°E, 23.15°N), and Hechi (108.05°E, 24.69°N). NcPSRV-1 infection was determined at the individual levels and more than 30 individuals were tested for each field population (Supplementary Table S3). The 5th instar nymph individuals were used for total RNA extraction. The cDNA was synthesized and qRT-PCR was performed as described above. The Ct value of NcPSRV-1 < 33, was regarded as infection. The infection rate of NcPSRV-1 in *N. cincticeps* field population was calculated by the number of infected individuals divided by the number of tested individuals.

### Detection of NcPSRV-1 in *N. cincticeps* Laboratory Populations

All laboratory populations of *N. cincticeps* reared on healthy rice plants were screened for NcPSRV-1 infection. The 5th instar nymph individuals from population A and B were tested for the presence of NcPSRV-1 in 2017 (*n* = 50). The infection rates of NcPSRV-1 in population A were examined again in 2018 (*n* = 48), 2019 (*n* = 52), and 2020 (*n* = 46), respectively. Total RNA extraction and cDNA synthesis of the 5th instar nymph individuals were performed as described above. The viral loads were quantified with qRT-PCR as described above. The prevalence of NcPSRV-1 was calculated as previously described (Detection of NcPSRV-1 in Field Populations of *N. cincticeps* section).

### Horizontal Transmission Detection

To investigate the role of honeydew in horizontal transmission route of NcPSRV-1, the presence of NcPSRV-1 was tested in the honeydew of *N. cincticeps*. The honeydew was excreted by *N. cincticeps* of population A on the field around the rice plants. After being exposed to the air more than 1 week, the honeydew crystals were formed, and then collected and dissolved in 100 μl RNAse-free water. The dissolved honeydew crystals were used for total RNA extraction and cDNA synthesis. NcPSRV-1 was detected using PCR as previously mentioned in Virus Detection and Quantification section.

To examine the horizontal transmission of NcPSRV-1 *via* the virus contaminated food, the 2nd instar nymphs of *N. cincticeps* from NcPSRV-1 negative population were fed on virus containing artificial diet at 25°C for 24 h. The artificial diet was a mix of 10% sucrose solution and crude virus filtrate at a ratio of 10:1. The 10% sucrose solution (W/V) was filtered through 0.22 μm filter (Millipore, MA, United States) under aseptic conditions. The crude virus filtrate was prepared using NcPSRV-1-infected 5th instar nymphs of *N. cincticeps*. After frozen in liquid nitrogen, 30–40 nymphs per 2.0 ml microcentrifuge tube were ground and 1 ml PBS was added into each tube. After resuspension, the homogenate was agitated at 4°C for 15 min and centrifuged at 4°C at 16,000 × *g* for 10 min. The supernatant was collected and subsequently filtered through 0.22 μm filter. Then the filtrate was concentrated using Ultra-15,100 kDa Centrifugal Filter tube (Millipore, Massachusetts, United States) and diluted to the preconcentration volume by adding 10% sucrose solution. This step was repeated twice and the crude virus filtrate was prepared. The feeding apparatus was a transparent tube (8.5 cm in diameter and 15 cm high), covered with two layers of stretched Parafilm (Neenah, WI, United States) sealing 2 ml artificial diet inside. The feeding apparatus was sterilized before use. During feeding, the apparatus was covered with black cloth except for the Parafilm area. After feeding for 24 h, the insects were transferred to fresh rice seedlings. Insects were collected 12 days postinoculation (dpi). The insects under the same conditions but without oral inoculation were used as a control. The filtrate of uninfected *N. cincticeps* was prepared with the same method. PCR and qRT-PCR were conducted for the virus detection as described above.

### Vertical Transmission Detection

Newly emerged individuals from NcPSRV-1 negative population and positive population (population A) were used to investigate the transmission modes of NcPSRV-1. The pairs of ♀+/♂+, ♀+/♂−, ♀−/♂+ and ♀−/♂− were mated and NcPSRV-1 infection rates of the 5th instar offspring were detected using qRT-PCR and PCR.

To confirm the potential vertical transmission routes (transovarial or transovum), NcPSRV-1 infections in eggs were further analyzed. The eggs with red eyespot (7 days) collected from population A were transferred into the sterile cell strainer and submerged in 100 ml of 1–2.33% sodium hypochlorite for 10 min to eliminate potential external viral contamination. The bleach solution was pipetted up and down until the eggs sank to the bottom of the strainer. Then the eggs was lifted from the bleach solution and washed with running water for 3 min followed by washing twice with 100 ml sterile deionized water. After thorough rinse, the eggs were air-dried and transferred to sterile 2.0 ml centrifuge tubes. Three replicates of sodium hypochlorite-treated eggs (*n* = 70 eggs per replicate) were tested, three replicates of the non-treated eggs (*n* = 70 eggs per replicate) were used as controls. To investigate the viral multiplication in eggs, the eggs without red eyespot (3 days after oviposition) were collected in triplicate (*n* = 70 eggs per replicate) from population A to compare NcPSRV-1 loads with the eggs with red eyespot (7 days after oviposition). The eggs with and without eyespot are shown in Supplementary Figure S1. NcPSRV-1 infections were quantified with qRT-PCR as previously described.

### Host Range of NcPSRV-1

Two closely related species of *N. cincticeps*, namely, *N. apicalis* and *R. dorsalis*, were tested as the alternative hosts for NcPSRV-1. The 5th instar nymphs of these two leafhopper species from labarotory populations were used to detect NcPSRV-1 presence.

The 2nd instar nymphs of *N. apicalis* and *R. dorsalis* were fed on NcPSRV-1-containing artificial diet at 25°C for 24 h and then transferred to fresh rice seedlings as previously described (Horizontal Transmission Detection section). On the 12th dpi, leafhoppers of the two species were collected to test for the presence of NcPSRV-1, respectively.

Rice plants were also tested for the existence of NcPSRV-1. The 5th instar nymphs from NcPSRV-1 positive population A were fed on rice seedlings (age 10 days) for 48 h. On 12 dpi, the seedling surface was washed by 1–2.33% sodium hypochlorite and flushed with water. After thorough rinse, the seedlings were sampled. The non-feeding rice seedlings at the same-day age were sampled as a control. The total RNA extraction, the cDNA synthesis, and virus detection of all the samples mentioned in this section were conducted as described above and NcPSRV-1 existence was tested using both PCR and qRT-PCR.

### Detection of Co-infection With RDV

Fifty of the 5th instar nymph individuals from population C were tested for the presence of NcPSRV-1 and RDV. The population B, which was RDV negative, was used as a control. Total RNA extraction and cDNA synthesis of the samples were conducted as described above. NcPSRV-1 and the reference gene *beta-actin* were amplified in the qRT-PCR using the primers described above. RDV was quantified using RDV-specific primers RDV-S8-S/RDV-S8-A (Supplementary Table S1). The viral loads of NcPSRV-1 and RDV were calculated with the method described above.

### Statistical Analysis

All data were present as mean ± standard errors (SEM). For the qRT-PCR results of NcPSRV-1 distributions, data were analyzed using one-way ANOVA followed by Tukey’s multiple comparison test (*p* < 0.05). The mean of the NcPSRV-1/*beta-actin* mRNA ratio in the virus detection of population A in 2017 was compared with that in 2018, 2019, and 2020 by using Student’s *t*-test (^*^ *p* < 0.05, ^**^ *p* < 0.01, ^***^ *p* < 0.001), respectively. Student’s *t*-tests were also used to evaluate the virus abundance between two populations with or without carrying RDV, and NcPSRV-1 titers in different groups of *N. cincticeps* eggs. Variable homogeneity tests were performed before the Student’s *t*-tests. All statistical tests were performed using Data Processing System (DPS) software (version 16.05; Tang and Zhang, 2013).

## Results

### Genome Sequence Analysis of NcPSRV-1

Two virus genome-like sequences [9,149 nucleotides (nt) and 1,043 nt in length] were initially identified from the transcriptomes of RDV-uninfected and RDV-infected *N. cincticeps* salivary glands. The transcript levels of these two sequences were significantly downregulated in the RDV-infected transcriptomes compared to the RDV-uninfected one (Table 1). Protein BLAST search against the NCBI database revealed that these two sequences had highest similarity to Hubei picorna-like virus 29, with 43 and 55% of the sequence identities, respectively, and together covered 89.45% of the Hubei picorna-like virus 29 genome sequence. The genome-like sequences were amplified with PCR (Supplementary Figure S2) and RACE, sequenced, and named as NcPSRV-1.

**Table 1:** Information of the picorna-like sequences discovered in the transcriptomes of *Nephotettix cincticeps*.

Gene ID	RDV-infected read count	RDV-uninfected read count	Fold change (log2)	*p*	*p*adj	Gene length (nt)
c57866_g1	19.4704	3,489.4320	−7.4856	1.64E-85	4.05E-81	9,149
c57866_g2	6.3543	1,273.1570	−7.6465	1.05E-74	1.93E-70	1,043

The NcPSRV-1 genome (GenBank accession number MW197427) is 10,524 nt in length excluding the 3' poly(A) tail and is A/U rich (A-28.02%, G-21.82%, U-30.38%, and C-19.77%) similar to other iflaviruses. The genome contains a single ORF (nt 630–10,208). The ORF encodes one large polyprotein of 3,192 aa and was flanked by UTRs. The 5' UTR is 629 nt in length and contains 13 AUG triplets. The 3' UTR of NcPSRV-1 is 327 nt in length located at positions nt 10,209–10,524 and followed by a poly(A) tail.

### Genome Organization

**Figure 1:**
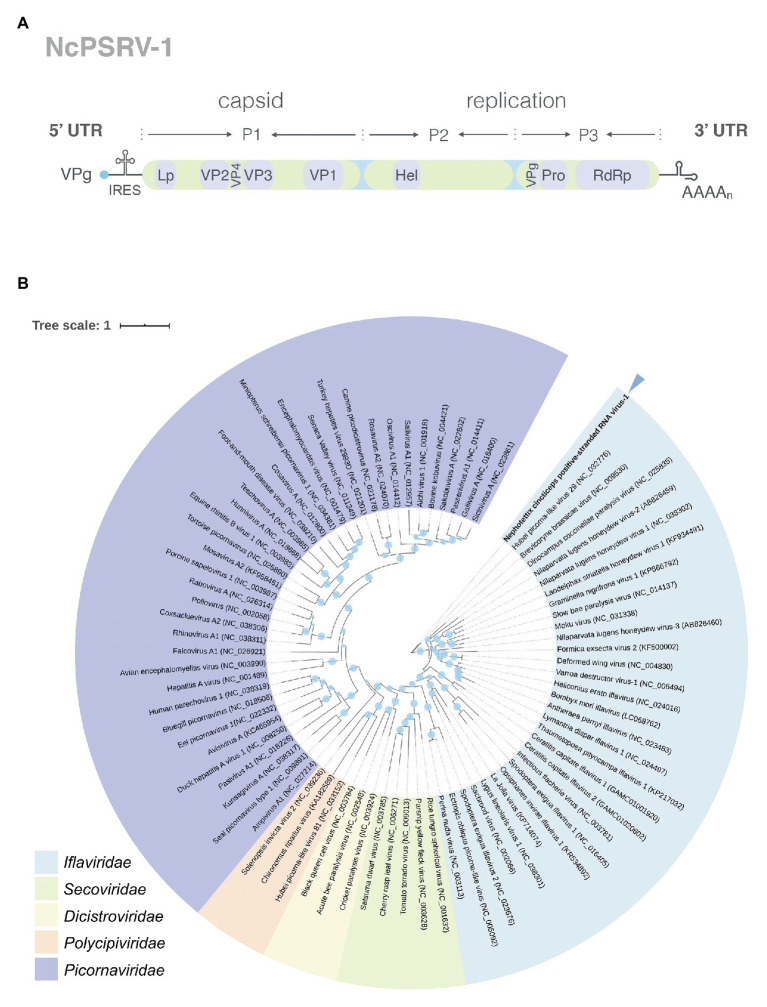
Analyses of NcPSRV-1. **(A)** Genome organization of NcPSRV-1. NcPSRV-1 genome encodes one polyprotein with the structural proteins VP1–VP4 (capsid proteins) located in the N-terminal region and the non-structural proteins responsible for replication and morphogenesis, helicase (Hel), protease (Pro), and RNA-dependent RNA polymerase (RdRp) in the C-terminal region. The open reading frame is flanked by 5' and 3' untranslated regions (UTRs), both contain secondary RNA structures. The viral genome-linked protein (VPg) is expected to link covalently to the 5' end of the genome and a leader protein (L) is also expected before the VP2 protein. **(B)** Phylogenetic analysis of NcPSRV-1. The phylogenetic tree was constructed based on the deduced RNA-dependent RNA polymerase domain. Viruses are from the order *Picornavirales* and the Genbank accession numbers are shown in each node. The sizes of the circles positioned on the branches represent the bootstrap values as percentages calculated from 1,000 replicates. NcPSRV-1 is shown in bold and indicated with an arrow.

Conserved domain analysis revealed NcPSRV-1 shared a typical iflavirus gene organization (Figure 1A). The structural proteins are located at the N-terminal of the polyprotein while the non-structural proteins follows immediately downstream in the C-terminal region.

**Figure 2:**
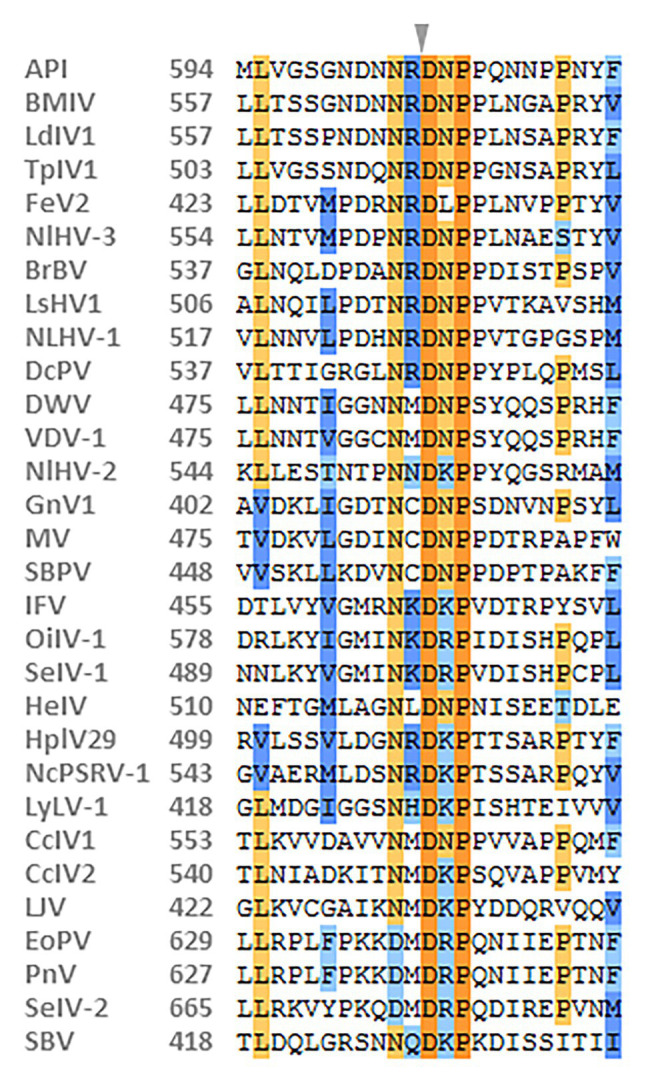
Alignment of the predicted cleavage sites of VP4-VP3 proteins of NcPSRV-1 among the iflaviruses. The amino acids shaded in orange and blue indicate the conservative and similar residues, respectively. The expected cleavage site is marked with an arrowhead. Numbers in the left of the sequences refer to the amino acid positions from the N-terminus of the viral polyprotein. The viral abbreviations and accession numbers are listed in Supplementary Table S2.

The capsid proteins of iflavirus arrange in the order VP2-VP4-VP3-VP1, and are often preceded by a short leader protein (L; Valles et al., 2017). Three capsid proteins were identified between residues 299–529, 554–806, and 985–1,255, corresponding to VP2, VP3, and VP1, respectively. The conserved motifs NxNxFQxG, WxGxLx_3_FxFx_7_Gx_5_YxP, and FxRG which have been observed in picornaviruses capsid proteins VP2, VP3, and VP1, respectively (Liljas et al., 2002), are divergent in iflaviruses to some extent, according to the multiple sequence analysis of iflaviruses and previous study (Murakami et al., 2013; Wu et al., 2019). Based on the sequences of iflaviruses selected from ICTV Virus taxonomy profile, the conserved motifs in structural proteins for iflaviruses were proposed (Supplementary Figure S3). A disorder site was predicted at aa positions 222–251 upstream the VP2 domain. As a leader protein was expected at the N terminus of the polyprotein, the disorder site may be the boundary of the expected leader protein and VP2. The VP4-VP3 cleavage site (N_X_/D_X_P), which has been experimentally confirmed (Liljas et al., 2002; Ongus et al., 2004; Lanzi et al., 2006; Murakami et al., 2013), was found at aa position 554 adjoining the C-terminus of the VP2, suggesting that a very small VP4 was right between the VP2 and VP3. Besides, the alignment of VP4-VP3 cleavage sites in iflaviruses showed that the aa “D” and “P” were relatively conserved (Figure 2).

The non-structural proteins appear in the order helicase (Hel), protease (Pro), and RdRp from N- to C-terminus. The helicase domain was identified at residues 1,551–1,721 and belongs to the helicase superfamily 3. This superfamily is associated with NTP-binding and characterized by three conserved motifs: motif A (^1578^GxxGxGKS^1585^), motif B (^1623^Qx_5_DD^1631^), and motif C (^1667^KKx_4_Px_5_NSN^1681^), while the motif C of NcPSRV-1 is slightly different from the consensus sequence KGx_4_Sx_5_STN (Gorbalenya and Koonin, 1989; Koonin and Dolja, 1993; Cheng et al., 2013). The chymotrypsin-like cysteine protease domain (aa 2,435–2,630) resembles the 3C protease of picornaviruses, including a cysteine-protease motif (^2588^GxCG^2591^; Ryan and Flint, 1997) and a substrate-binding motif (^2605^GxHxxG^2610^; Gorbalenya et al., 1989). The RdRp domain belonging to RNA polymerase superfamily I (Kamer and Argos, 1984) was located at aa 2,677–3,159. All eight characteristic motifs of the RdRp domain were identified, including motif V [^2988^PSGx_3_Tx_3_N(S/T)^2,997^] and motif VI (^3035^YGDD^3038^; Koonin and Dolja, 1993). Besides, a highly conserved domain (^2779^TSxGxP^2785^) was found at the location prior to the motif I of RdRp domain (de Miranda and Genersch, 2010).

In addition, several other structures were also deduced. A coiled coil was predicted at aa 2,036–2,063 in the C-terminus of the P2 region. Two transmembrane regions were detected at aa 1,347–1,368 (VVMAIAVGSFLMSLGIVCFESL) and aa 2,290–2,307 (YKWIFVSAAAIIGGFFLAT), followed by 3C-protease proteolytic sites [AxPE/G (A/M)] AKPE^1395^/A and ANPE^2379^/M, respectively (Lanzi et al., 2006). These two proteolytic sites may indicate the separation of the helicase from the polyprotein. The NPG/P catalytic site, which has been found in many picornaviruses (Zell et al., 2017) and some iflaviruses such as DWV and VDV-1 (de Miranda and Genersch, 2010), is absent from the NcPSRV-1 sequence.

### Phylogenetic Analysis

Phylogenetic analysis was performed using the highly conserved RdRp region to investigate the relationships of NcPSRV-1 among the viruses in the order *Picornavirales*. Fifteen member species and 13 unclassified viruses in the family *Iflaviridae* and the type species in other families of *Picornavirales* were selected for analysis. NcPSRV-1 is clustered with the members of *Iflavirus* (Figure 1B), indicating that NcPSRV-1 could be classified into the family *Iflaviridae*. NcPSRV-1 is located next to Hubei picorna-like virus 29 and generally more closely related to the iflaviruses discovered from Hemiptera. In the phylogenetic analysis, viruses infecting the hosts of the same order segregate into different clades, suggesting that the iflaviruses do not strictly follow the evolutionary relationships with their hosts.

### Presence of NcPSRV-1 in Different Tissues and Developmental Stages of *N. cincticeps*

**Figure 3:**
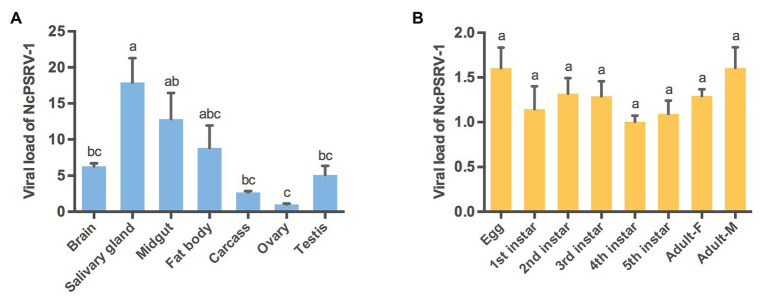
Tissue and developmental expression patterns of NcPSRV-1 in *N. cincticeps*. **(A)** The viral loads of NcPSRV-1 in *N. cincticeps* in brain, salivary gland, midgut, fat body, carcass, ovary and testis. **(B)** The viral loads of NcPSRV-1 of *N. cincticeps* in eggs, 1st-5th instar nymphs and adults (males and females). Data represent means ± standard errors (SEM; *n* = 3). Bars annotated with the same letters are not significantly different (*p* < 0.05, Tukey’s multiple comparison test).

The relative abundance of NcPSRV-1 in the brain, salivary gland, midgut, fat body, carcass, ovary, and testis of *N. cincticeps* was quantified with qRT-PCR. The results showed that NcPSRV-1 was present in all the tested tissues (Figure 3A). The transcripts of NcPSRV-1 were detected at the highest level in salivary gland followed by midgut and fat body (*F* = 6.8360, *p* = 0.0015). The low viral loads were observed in the brain, testis, carcass, and ovary. The viral load in testis was higher than ovaries.

The relative abundance of NcPSRV-1 in eggs, 1st-5th instar nymphs, females and males of *N. cincticeps* was determined using qRT-PCR. The viral titers were at the similar levels and exhibited no significant differences among the different developmental stages (*F* = 1.4860, *p* = 0.2413). These results indicate that NcPSRV-1 is present throughout all developmental stages of *N. cincticeps* at a relatively stable viral level (Figure 3B).

### Prevalence of NcPSRV-1 From Different Geographical Regions

**Figure 4:**
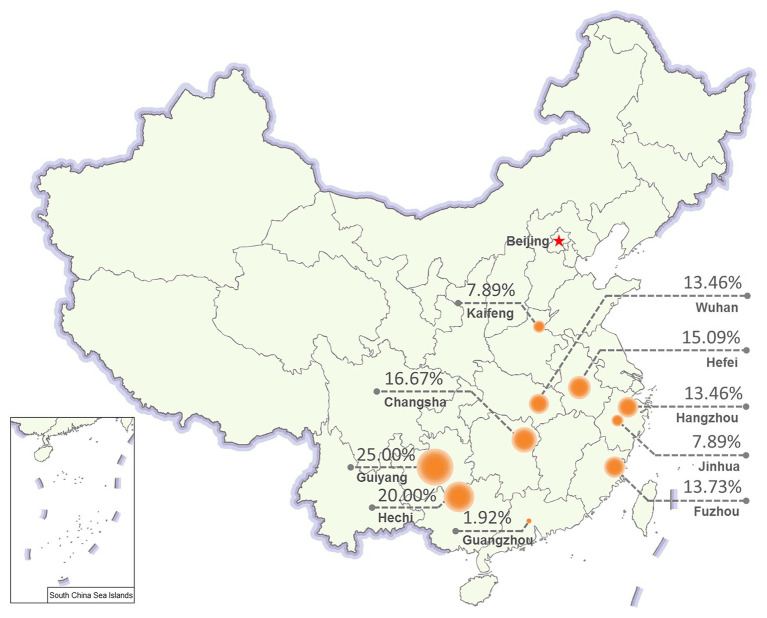
Prevalence of NcPSRV-1 in different geographical regions. Ten *N. cincticeps* field populations were collected from different geographical regions in China. The NcPSRV-1 infection rate of each population is shown (*n* ≥ 38).

To evaluate the prevalence of NcPSRV-1, *N. cincticeps* field populations were collected from 10 different locations in China as follows: Hangzhou, Jinhua, Wuhan, Hefei, Guiyang, Fuzhou, Changsha, Kaifeng, Guangzhou, and Hechi in 2017. The 5th instar nymph individuals from each of the field populations were examined for the infection rate of NcPSRV-1 with qRT-PCR. All the field populations were NcPSRV-1 positive. The infection rates were ranging from 1.92 to 25.00% (Figure 4 and Supplementary Table S3A). The infection rates of Hechi and Guiyang populations were higher than others, while Guangzhou population displayed the lowest. These data demonstrated that NcPSRV-1 was present in the field populations of *N. cincticeps* among the rice regions of the Southwest, Southeast, and Central China.

### Viral Abundance in Laboratory Populations

**Figure 5:**
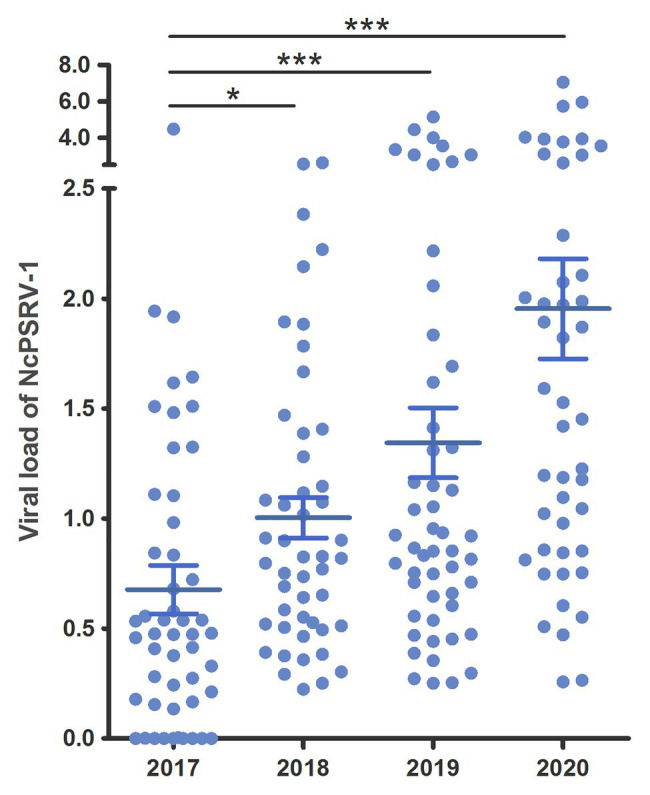
Viral loads of NcPSRV-1 in laboratory populations. The viral loads of population A in 2017–2020 are shown individually. Each spot represents the value for an individual. The average viral levels of population A in each year represent means ± SEM (*n* ≥ 46). Statistical significance is indicated by asterisks (^*^ *p* < 0.05, ^***^ *p* < 0.001, Student’s *t*-test).

Populations A and B were maintained in our lab since 2014/2015 and were tested for the presence of NcPSRV-1 in 5th instar nymph individuals in 2017. Unexpectedly, both populations were NcPSRV-1-positive with relatively high infection rates (56–100%; Supplementary Table S3B) compared with the field populations (1.92–20.00%; Supplementary Table S3A). In order to investigate the tendancy for NcPSRV-1 abundance in *N. cincticeps* laboratory population, the infection rates of population A were tested again in 2018 and 2019. The average viral load of NcPSRV-1 in 2017 was significantly different with the result of 2018 (*t* = 2.2674, *df* = 93.80, *p* = 0.0257), 2019 (*t* = 3.4409, *df* = 90.03, *p* = 0.0009), and 2020 (*t* = 5.1243, *df* = 65.55, *p* = 0.0001; Figure 5), indicating a clear trend of increasing in *N. cincticeps* laboratory population A, not only the number of infective 5th instar nymphs but also the average viral load in population A. Worth noting, the viral infection rates of population A in 2018, 2019, and 2020 reached 100% (Supplementary Table S3B).

### Horizontal Transmission Detection

**Figure 6:**
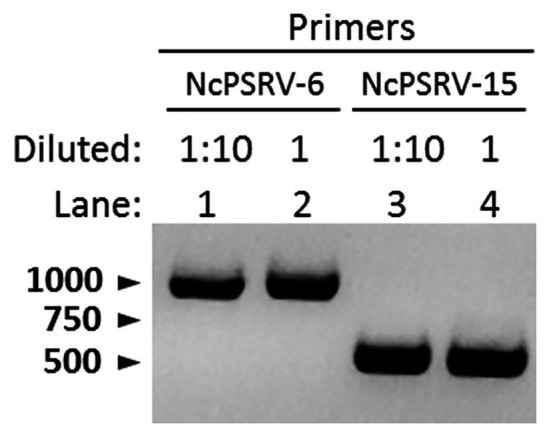
Detection of NcPSRV-1 in the honeydew of viruliferous *N. cincticeps*. NcPSRV-1 was detected *via* PCR by using two pairs of specific primers NcPSRV-6F/NcPSRV-6R and NcPSRV-15F/NcPSRV-15R. In lanes 1 and 3, the cDNA sample from the honeydew used for PCR was diluted 10 times. The cDNA sample used in lanes 2 and 4 was not diluted.

The honeydew of *N. cincticeps* in population A was examined for NcPSRV-1 presence. Intense and specific bands of expected sizes were observed in NcPSRV-1 detection (Figure 6), indicating NcPSRV-1 existed in the honeydew of virus positive *N. cincticeps* population. The honeydew used in this study had been exposed to air for more than a week, the result demonstrated that NcPSRV-1 could be preserved in the honeydew for at least a week.

The NcPSRV-1 negative nymphs of *N. cincticeps* were fed on the artificial diet containing crude virus extract prepared from NcPSRV-1 positive nymphs. After 12 dpi, NcPSRV-1 was detected and at least 92.11% of the tested *N. cincticeps* was virus positive while NcPSRV-1 was absent in control *N. cincticeps* fed with the artificial diet without crude virus extract (Supplementary Figure S4). This result provided strong evidence that NcPSRV-1 could be horizontally transmitted among the host *N. cincticeps* effectively.

### Vertical Transmission Detection

To ascertain the vertical transmission modes of NcPSRV-1, offspring from four mated pairs (♀+/♂+, ♀+/♂−, ♀−/♂+, and ♀−/♂−) were examined for the virus infection. NcPSRV-1 could be vertically transmitted from the mated pairs ♀+/♂+ and ♀+/♂−. The transmission efficiency attained 100% among the offspring of the infected females and males, higher than 80% in the mated pairs ♀+/♂− (Table 2). When the females were infected, the infected males could elevate the infection rate of NcPSRV-1 in the offspring (Supplementary Figures S5A–C). In case of uninfected females, whether the males were infected or not, the offspring were all NcPSRV-1 negative. These results suggested that virus-infected females determined the vertical transmission of NcPSRV-1.

**Table 2:** Vertical transmission efficiency of *Nephotettix cincticeps* positive-stranded RNA virus-1 (NcPSRV-1).

Mated pairs	Number of tested *N.cincticeps*	Transmission efficiency (%)
♀+/♂+	30	100
♀+/♂−	10	80
♀−/♂+	18	0
♀−/♂−	30	0

Infected individuals = “+”; uninfected individuals = “−”.

**Figure 7:**
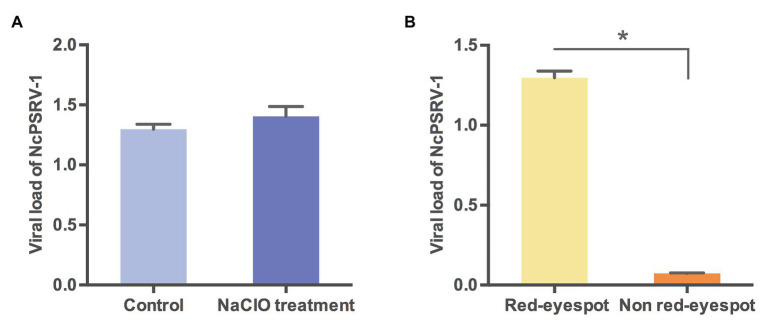
Viral loads of NcPSRV-1 in *N. cincticeps* eggs. **(A)** The viral loads of NcPSRV-1 in sodium hypochlorite-treated eggs and non-treated eggs. **(B)** The viral loads of NcPSRV-1 in the eggs with red eyespot (7 days after oviposition) and without red eyespot (3 days after oviposition). Means ± SEM (*n* = 3). Statistical significance is indicated by asterisks (^*^ *p* < 0.05, Student’s *t*-test).

The vertical transmission routes were further examined to explore whether NcPSRV-1 was transmitted *via* transovum (on the surface of eggs) or transovarial (within the eggs) way. No significant difference was observed between the sodium hypochlorite-treated eggs and the non-treated eggs (*t* = 1.1783, *df* = 4, *p* = 0.3040; Figure 7A), indicating that the vertical transmission of NcPSRV-1 mainly occurred transovarially. Moreover, the eggs with red eyespot (7 days after oviposition) had much higher viral load of NcPSRV-1 than the eggs without red eyespot (3 days after oviposition; *t* = 29.288, *df* = 2.035, *p* = 0.0011; Figure 7B), implying the rapid viral proliferation in the infected eggs.

### Host Range of NcPSRV-1

To determine the potential host range of NcPSRV-1, the 5th instar nymphs of *N. apicalis* (*n* = 35) and *R. dorsalis* (*n* = 36) from labarotory populations were tested for the existence of NcPSRV-1. The results showed that no virus positive individuals were detected in the nymphs from these two species (Table 3 and Supplementary Figures S6A,C).

**Table 3:** Host range detection of NcPSRV-1.

Species	Viral inoculation	Number of tested individuals	Infection rate (%)
*Nephotettix apicalis*	−	35	0.00
	+	30	3.33
*Recilia dorsalis*	−	36	0.00
	+	30	0.00
*Oryza sativa* (variety TN1)	−	30	0.00
	+	30	0.00

Artificial diet without crude virus = “−”; artificial diet with crude virus = “+”.

The NcPSRV-1 negative nymphs of *N. apicalis* and *R. dorsalis* labarotory populations were then fed on artificial diet containing crude virus extract to inoculate NcPSRV-1. After 12 dpi, an infection rate of 3.33% was detected in *N. apicalis* (Table 3 and Supplementary Figure S6B) while no NcPSRV-1 was detected in *R. dorsalis* (Table 3 and Supplementary Figure S6D). Moreover, NcPSRV-1 could not be detected in the rice seedlings with or without feeding by *N. cincticeps* (Table 3 and Supplementary Figures S6E,F), indicating that NcPSRV-1 in insect body may not derive from rice plants.

### Co-infection With RDV

**Figure 8:**
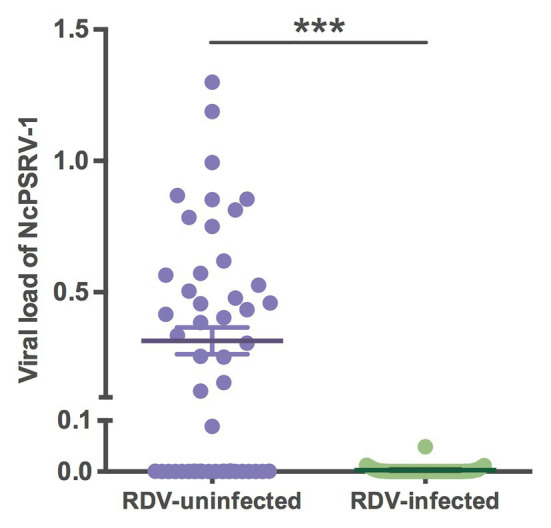
Viral loads of NcPSRV-1 in the rice dwarf virus (RDV)-feeding and non-feeding *N. cincticeps* populations. Each spot represents the value for an individual. The average viral levels of population B (*n* = 50) and C (*n* = 42) represent means ± SEM. Statistical significance is indicated by asterisks (^***^ *p* < 0.001, Student’s *t*-test).

To assess the impact of RDV on NcPSRV-1, the viral loads of RDV and NcPSRV-1 in population B (*n* = 50) and C (*n* = 42) were determined. The infection rate of RDV was 45.24% in population C, while NcPSRV-1 was present at 9.52%. In the RDV negative population B, 56.00% individuals were NcPSRV-1 positive. The viral loads in populations B and C were significantly different (*t* = 6.0883, *df* = 49.06, *p* = 0.0001; Figure 8) which appear that NcPSRV-1 has divergent levels in the RDV-uninfected and RDV-infected *N. cincticeps* populations. The result was in accordance with the differential expression analysis in Table 1, in which the RDV infection rate of *N. cincticeps* population D used for RDV-infected transcriptome analysis was more than 90%. The differential expression analysis in Table 1 was also verified using qRT-PCR (data not shown). Given the low infection rates of NcPSRV-1 among field populations in this study, the results implicate that RDV may antagonize NcPSRV-1 proliferation in the populations of low NcPSRV-1 prevalence, due to the fact that the RDV-infected *N. cincticeps* populations were reared on RDV-infected rice plants immediately after captured from the fields before any NcPSRV-1 inoculation.

## Discussion

Owing to high-throughput RNA sequencing technology, the number of identified iflaviruses rapidly increased in recent years. In this study, we identified a novel virus NcPSRV-1 from *N. cincticeps* transcriptomes. Genome organization, sequence alignments, and phylogenetic analysis revealed that NcPSRV-1 is a member of the family *Iflaviridae*. Thus, NcPSRV-1 was the first iflavirus discovered in *N. cincticeps*, expending our knowledge of virus prevalence, abundance, and distribution *in vivo*.

In previous studies, the iflavirus capsid proteins were compared with the conserved motifs observed in the family *Picornaviridae*. Therefore, we attempted to summarize sets of conserved motifs from iflavirus capsid proteins, which are more appropriate for iflaviruses (Supplementary Figure S2). Pairwise comparisions revealed that the VP1 capsid proteins were more variable than VP2 and VP3 in iflaviruses and seemed to have evolved more independently from the others. For the conserved motifs in VP1, especially motif I, II, and III, were missing among some viruses, thus the conserved motifs in VP1 we proposed in this work cannot be applied to every iflaviruses, but the missing parts of the motifs may provide some basis for the taxonomy of the genus *Iflavirus*. In the phylogenetic analysis based on RdRp domain, iflaviruses did not strictly co-evolve with their hosts. In the aa alignments of iflaviruses, sequence similarities were not highly correlated with their host’s evolutionary relationships. Taken the alignment of VP1, for example, Antheraea pernyi iflavirus (API), Bombyx mori iflavirus (BMIV), Lymantria dispar iflavirus 1 (LdIV1), Heliconius erato iflavirus (HeIV), and Thaumetopoea pityocampa iflavirus 1 (TpIV1) discovered in Lepidoptera insects had identical conserved motifs, sharing high similarities in the capsid protein sequences, while Lepidoptera-infecting iflaviruses Ectropis obliqua picorna-like virus (EoPV), Perina nuda virus (PnV), IFV, Opsiphanes invirae iflavirus 1 (OiIV-1), Spodoptera exigua iflavirus 1 (SeIV-1), and Spodoptera exigua iflavirus 2 (SeIV-2) were similar to each other but presented low degrees of sequence conservation compared to the viruses mentioned above, even though BMIV and IFV infected the same host *Bombyx mori*. The variation in VP1 may be due to the differences lying in the pathogenicity, virulence, and host range. Whether there is a direct linkage between the pathogenicity and the sequence variation in VP1 needs further verification.

NcPSRV-1 presented in all tested tissues, being most abundant in the salivary glands and displayed the least in the ovaries. NcPSRV-1 titers in different life stages of *N. cincticeps* were relatively stable, exhibiting the adaptability to the insect host. Like NcPSRV-1, many iflaviruses appeared to be distributed in multiple tissues and could be detected in multiple life stages of the hosts, however, the spatial distribution tendency of iflaviruses varied. The crippled bees were strongly DWV-positive in all body parts, while the asymptomatic bees were never virus positive in the head (Yue and Genersch, 2005). The Helicoverpa armigera iflavirus (HaIV) titers were higher in fat body than the other tissues (Yuan et al., 2017). SeIV-1 was most abundant in the midgut, the viral titer of midgut was 5- or 10-fold relative to the hemocyte and fat body (Millan-Leiva et al., 2012). IFV, found in *B. mori*, was primarily described to target the goblet cells of the midgut epithelium (Isawa et al., 1998). To our knowledge, NcPSRV-1 was the first iflavirus described to be most abundant in salivary glands. The tissue distribution may associate with the virus functions. *N. cincticeps* is a piercing-sucking insect, secreting saliva while feeding in phloem. NcPSRV-1 accumulated in the salivary glands may be feasible to be secreted with the saliva.

High infection rates of NcPSRV-1 were observed in *N. cincticeps* laboratory populations. NcPSRV-1 could long exist in the laboratory populations of *N. cincticeps* with the increasing viral loads and infection rates. After rearing in the laboratory conditions for 3 years, the infection rates of *N. cincticeps* population by NcPSRV-1 were maintained around 100%. High prevalence also occurred among other iflaviruses. In several detection which was examined at approximately 6-month intervals, NLHV-2 was detected in 80–100% and NLHV-3 in 60–100% of the *Nilaparvata lugens* colony (Murakami et al., 2014). After rearing in greenhouse for several generations, the *Laodelphax striatellus* colony was 100% LsIV1-infected (Wu et al., 2019). In the case of Ceratitis capitata Iflavirus 2 (CcaIV2), 100% *Coleomegilla capitata* individuals from the V8G and V8A strains and 77% of the V8 strains were carrying CcaIV2 at a high titer (Llopis-Gimenez et al., 2017).

NcPSRV-1 infections did not manifest evident pathogenic symptoms and damages to *N. cincticeps* hosts throughout all life stages and populations by far. NcPSRV-1 appeared to lead a persistent, covert infection in the host, even at a high viral level. Several iflaviruses have been brought to the notice due to the overt infections in the infected insects. For instance, sacbrood bee virus (SBV), the first reported bee virus, made the honeybee larvae sac-like and failed to pupate and eventually died at the last larval stage (Grabensteiner et al., 2001; Shen et al., 2005a). IFV can cause flacherie disease of *B. mori* (Isawa et al., 1998). SBPV linked to high mortality of colonies when infested with *V. destructor* (Carreck et al., 2010). DWV caused wing deformities and premature death in adult honeybees (Chen et al., 2005; de Miranda and Genersch, 2010). Additionally, Dinocampus coccinellae paralysis virus (DcPV) was reported to induce a severe neurological disorder through replicating in the cerebral ganglia of the host *Coleomegilla maculata* (Dheilly et al., 2015). HaIV infection significantly delayed the host developmental time both in larval and pupal periods and decreased the pupation rate. Besides, HaIV resulted in a 51.6% weight reduction on day 8 and higher mortality in larvae (Yuan et al., 2020). SeIV-1 infection led detrimental effects to the *Spodoptera exigua* host in respects of reductions of larval weight gain and female adult proportion (Carballo et al., 2020). In contrast, covert infections seemed more general among iflaviruses. Some inapparent iflavirus infections may be deleterious to the hosts but not lethal so they have not drawn enough attention yet. In the absence of *Varroa*, DWV and SBPV can persist in naturally covert infections in the honeybees (Carreck et al., 2005; Yue and Genersch, 2005). SBV also existed in the adult bees as a latent infection (Grabensteiner et al., 2001; Shen et al., 2005a). According to the references reported, no obvious symptoms and negative effects caused by iflaviruses were described in Hemiptera and Diptera hosts to date. For instance, no visible symptoms were detected among NlHV-1, NlHV-2, and NlHV-3-infected colonies (Murakami et al., 2013, 2014). LsIV1 could not influence the biological features such as development and reproduction of *L. striatellus* (Wu et al., 2019). No statistically significant correlation between the viral abundance of CcaIV2 and the male host fitness were observed with respect to mating performance, flying performance, and lifespan (Llopis-Gimenez et al., 2017). Additionally, iflaviruses may be beneficial to the hosts. For example, BrBV, which appears to not be lethal to laboratory aphids, however, when accumulated at a high level, makes the adult aphids less attractive to parasitoid wasps in the natural population (Ryabov, 2007).

Horizontal transmission commonly occurs in the ingestion of virus contaminated food, and among social insects, trophallaxis contributes to the virus dispersal as well (Yue and Genersch, 2005; de Miranda and Genersch, 2010). DWV, the most studied among iflaviruses for the transmission route, could be transmitted between *Apis mellifera* horizontally *via* fecal-cannibal-oral and nursing bee contact (Chen et al., 2006a; Yue and Genersch, 2005), vector-mediated transmission induced by *Varroa* mite (Wilfert et al., 2016), and sexual transmission (Amiri et al., 2016). NLHV-1 was reported to transmitted through honeydew which were excreted from the viruliferous *N. lugens* (Murakami et al., 2013). In the present study, horizontal transmission of NcPSRV-1 was validated *via* the oral inoculation experiment with a relatively high infection rate. NcPSRV-1 was confirmed to be excreted in the honeydew of virus positive population, and could be preserved for at least a week under the laboratory conditions. Like NLHV-1, the virus-containing honeydew may be an inoculum of NcPSRV-1 for *N. cincticeps*, as the insects could be inoculated through the surface of rice plants contaminated with honeydew during the stylet penetration (Murakami et al., 2013). The sticky and sugar-rich honeydew could make the virus adhere to plant surface and may protect this single-stranded RNA virus from degradation, thereby increasing the horizontal transmission efficiency of NcPSRV-1. However, this hypothesis needs to be further ascertained. Vertical transmission of NcPSRV-1 was supported by the crossing experiments between NcPSRV-1 positive and negative *N. cincticeps*. Slightly different from the reported model of iflaviruses, the vertical transmission of NcPSRV-1 was female-dependent in *N. cincticeps*. Covert DWV infections could be vertically transmitted from DWV-infected queens and drones to their offspring through fertilized and unfertilized DWV-positive eggs, as well as DWV-positive semen (Chen et al., 2006b; Yue et al., 2006, 2007; de Miranda and Fries, 2008). As to NcPSRV-1, when the *N. cincticeps* females were infected, NcPSRV-1 could be transmitted to the offspring with high efficiency and the infected males could promote the infection rate of their offspring. Whereas, when the females were negative, the infected males lacked the ability to transmit NcPSRV-1 to their offspring. In the case of LsIV1, the infected *L. striatellus* female had similar high transmission efficiency, but the LsIV1 infected male had 50% infected offspring (Wu et al., 2019) which was quite different from NcPSRV-1. Transovarial infection route of NcPSRV-1 was also verified. Unlike HaIV (Yuan et al., 2017), NcPSRV-1 was mostly distributed within the eggs rather than on the surface. Thus, NcPSRV-1 was mainly vertically transmitted *via* maternal lines. Furthermore, the virus loads in eggs suggested rapid viral replication occurred and the low virus level in early eggs strongly indicated the possibility of the existence of egg or ovarian membrane barrier. However, the membrane barrier needs to be further elucidated.

The closely related species of *N. cincticeps*, *N. apicalis* and *R. dorsalis* were investigated as the potential hosts for NcPSRV-1. The virus was capable of infecting *N. apicalis* with a rather low infection rate but could not be detected in *R. dorsalis*. The results implied that these two leafhopper species may not serve as the natural hosts of NcPSRV-1. Meanwhile, NcPSRV-1 was absent in the rice 12 days postinoculation, indicating that the virus was unable to propagate in rice plants. NcPSRV-1 may infect *N. apicalis* and *R. dorsalis* at a relatively low level of virion production, but due to the limitation in sensitivity and accuracy of the detection methods, it has a possibility that the virus could not be detected. Moreover, the infection capacity of NcPSRV-1 in insects may differ between the laboratory populations and the field populations, and also differ in different life stages of *N. cincticeps*, but in this study, only 5th instar nymphs from the laboratory populations were tested. These may be the reason for failed detection. Beyond the original host, which the viruses derived from, the host range among most iflaviruses was poorly understood and some iflaviruses appeared to have a narrow host range (Valles et al., 2017). LsIV1 was reported to be present in *L. striatellus* but negative in other planthoppers, such as *N. lugens* and *S. furcifera*, and the rice seedling on which the *L. striatellus* had been reared for 2 weeks (Wu et al., 2019). SeIV-1 found in *S. exigua*, failed to be recognized in the *Spodoptera frugiperda* laboratory colony which had been reared together with the *S. exigua* colonies over 20 generations (Millan-Leiva et al., 2012). However, several viruses have been reported to produce cross-species transmission and infect a wide range of hosts. For instance, analyses of four stink bugs of different species revealed the presence of HhV (Dos Santos et al., 2019). DWV infects several honeybees (mainly *A. mellifera*; de Miranda and Genersch, 2010; Ellis and Munn, 2015), bumble bees (Genersch et al., 2006), and also a small hive beetle, *Aethina tumida*, which is a scavenger and parasite of honeybee colonies (Eyer et al., 2008). Crucially, DWV infects *Varroa* and has been transmitted worldwide driven by the spread of the *Varroa* mites (Bowen-Walker et al., 1999; Shen et al., 2005b; Chen et al., 2006a; Wilfert et al., 2016). SBV and SBPV could also infect honeybees and the ectoparasitic mite *Varroa destructor* (Tentcheva et al., 2004). MV could be possibly transmitted from the origin host *Vespula pensylvanica*, a predatory social wasp, to honeybees or vice versa and then onto *Varroa* (Carreck et al., 2010; Mordecai et al., 2016b). In view of honeybee viruses DWV, SBPV, and SBV which can be vectored by parasitic mites, the parasitic invertebrates in the same ecological niche with *N. cincticeps* require further investigation for their role in viral transmission.

In the present study, NcPSRV-1 could be co-infected with NcNSRV-1 (negative single-stranded RNA, unpublished) and RDV (double-stranded RNA virus) in the fields or laboratory populations, respectively (Supplementary Tables S3B,C). Especially, RDV seemed to have an antagonistic effect on the NcPSRV-1 infection. When sustaining continuous RDV infection, NcPSRV-1 was detected at low infection rate, while the infection rates and vial titers of NcPSRV-1 kept increasing as the *N. cincticeps* hosts were reared on the RDV-uninfected rice plants under the same conditions. Three hypotheses are proposed: (i) RDV may compete with NcPSRV-1 for the resources used for viral replication in the cytoplasm of the infected cells; (ii) the immune response triggered by RDV in *N. cincticeps* may affect the proliferation of NcPSRV-1; (iii) the alteration in nutrition or immune signaling pathway caused by continuous infection of RDV in rice may influence the insect host, therefore, impacting on the replication of NcPSRV-1. Whether there was a direct or indirect interaction between NcPSRV-1 and RDV require further investigation. Iflaviruses are often found co-infected with other pathogens. The honey bee, *A. mellifera*, and ectoparasitic mite, *V. destructor*, are commonly infected by multiple viruses, including IAPV, BQCV, DWV, and SBV associated with colony collapse disorder (CCD; Carrillo-Tripp et al., 2016; Mordecai et al., 2016a). Several iflaviruses, e.g., EoPV, PnPV and SeIV-2, exist simultaneously with baculoviruses in lepidopteran pests (Wang et al., 1999, 2004; Jakubowska et al., 2014). Cases of the interactions between iflaviruses and the co-infected pathogens have been reported. The presence of SeIV-1 in association with Spodoptera exigua multiple nucleopolyhedrovirus (SeMNPV) could negatively affect the SeMNPV pathogenicity. Meanwhile, the viral association increased the infectivity of SeIV-1 and elevated its resistance to UV-C and high temperature as the SeIV-1 embedded in the polyhedrin matrix of SeMNPV (Jakubowska et al., 2016). When infected with SeIV-1 and SeIV-2, the *S. exigua* larvae were clearly more susceptible to SeMNPV, with a nearly 4-fold reduced LC_50_ and a 12 h decrease in mean time to death (MTD) of SeMNPV (Carballo et al., 2020). The reported occurrence of virus-pathogen co-infection was only the tip of the iceberg where must be massive complex interaction nets behind, including virus-host and virus-pathogen interactions. This reminds us when focusing on a certain virus, the potential background infections should be taken into account (Dapalma et al., 2010; Carrillo-Tripp et al., 2016). And also, the exploration of virus-virus interactions may provide new strategy for rice dwarf disease control.

Taken together the characteristics of NcPSRV-1, extensive geographical distribution, sustainable increasing infection rates in population of high density, covert and persistent infection, horizontal and vertical transmissions, and systematic infection, we think NcPSRV-1 or iflavirues of the similar traits has the great potential to be engineered. For instance, the iflavirues may be modified to deliver genes of interest. The positive-sense single-strand RNA virus genome, which serves as mRNA for the IRES-mediated translation of polyprotein after release, could be reformed as a vector carrying target genes. The target genes could be replicated and translated simultaneously with the engineered viral genome during the viral replication cycle and released into the cytoplasm by cleavage of the 3C^pro^ and intermediate 3CD^pro^ (Jiang et al., 2014) with the proteolytic process. Eventually, the newly generated viral genome could be encapsulated into the virions and ready for next life cycle. The IRES of the iflaviruses could also be integrated into a vector as an element to mediate (improve or suppress) the expression level of the target genes. Taking advantage of the good adaptability, an iflavirus that cause persistent covert infection in the pest host, could be combined with other virulent virus to generate the recombinant pathogenic virions which would lead to the death of the pests for the purpose of precision pest control. However, these hypotheses mentioned above need further substantiation.

Based on the iflaviruses reported previously, we speculate iflaviruses exist universally in insects and many of them are mutual symbiotic or in harmony with their hosts without causing obviously damage to host fitness. The insect iflaviruses may be invertebrate-specific and widespread in the field populations. These traits may indicate that the iflaviruses infecting asymptomatically may be a part of the host virome. Furthermore, insect iflaviruses co-infect with diverse pathogens, and several cases of iflavirus-pathogen interactions have been reported. Iflaviruses could even affect the behavior of the hosts or the parasitoid wasps of the hosts. Insects, especially those in Hemiptera and Diptera, are the natural vectors of numerous pathogenic viruses and of great importance to agriculture and public health. All these properties make insect iflaviruses a reservoir for manipulating insect vectors or the route of pathogen transmission. The further study of iflaviruses will provide us new perspective and open up new possibilities for the integrated pest management in the future.

## Conclusion

A novel positive-sense single-stranded RNA virus named NcPSRV-1 was identified and characterized in this study. The virus genome is 10,524 nt in length encoding one large polyprotein and shares a typical iflavirus genome arrangement. Multiple sequence alignment and phylogenetic analysis showed that NcPSRV-1 has high sequence similarity with Hubei picorna-like virus 29 and clusters with the members of the genus *Iflavirus*. NcPSRV-1 was abundant in salivary glands and propagated stably in all life stages of *N. cincticeps*. NcPSRV-1 was wide spread in the fields populations and could maintain a relatively high infection rate when the host *N. cincticeps* was reared at high density. NcPSRV-1 persisted in a covert infection in the host and could be transmitted both horizontally and vertically. Co-infection with RDV was discovered and RDV seemed to have an antagonistic effect on the NcPSRV-1 infection.

## Data Availability Statement

The datasets presented in this study can be found in the GenBank, NCBI. Accession numbers are listed in the Supplementary Table S2.

## Author Contributions

WJ performed the experiment and wrote the manuscript. JL assisted in performing partial experiments. HY, JL, and XC assisted the sample collection. YY assisted the phylogenic reconstruction. GY, YB, and QS conceived the experiments. FW, YB, QS, and GY revised the manuscript. All authors contributed to the article and approved the submitted version.

### Conflict of Interest

The authors declare that the research was conducted in the absence of any commercial or financial relationships that could be construed as a potential conflict of interest.

## Footnotes

^1^https://www.ncbi.nlm.nih.gov/orffinder/

^2^http://rna.tbi.univie.ac.at/cgi-bin/RNAWebSuite/RNAfold.cgi

^3^http://unafold.rna.albany.edu/?q=mfold/download-mfold

^4^https://blast.ncbi.nlm.nih.gov/Blast.cgi

^5^http://www.ebi.ac.uk/interpro/

^6^https://viralzone.expasy.org/294

^7^https://talk.ictvonline.org/ictv-reports/ictv_online_report/positive-sense-rna-viruses/picornavirales/w/iflaviridae

^8^https://itol.embl.de/
