# Supplementary material for: A Novel Iflavirus Was Discovered in Green Rice Leafhopper Nephotettix cincticeps and Its Proliferation Was Inhibited by Infection of Rice Dwarf Virus
Source: Front Microbiol. 2021 Jan 8;11:621141. doi: 10.3389/fmicb.2020.621141 (PMC7820178; doi:10.3389/fmicb.2020.621141)
Supplement: Supplementary file 1 [file Data_Sheet_1.zip › Supplementary Material Presentation/Supplementary Figure S3.pdf]

A VP2

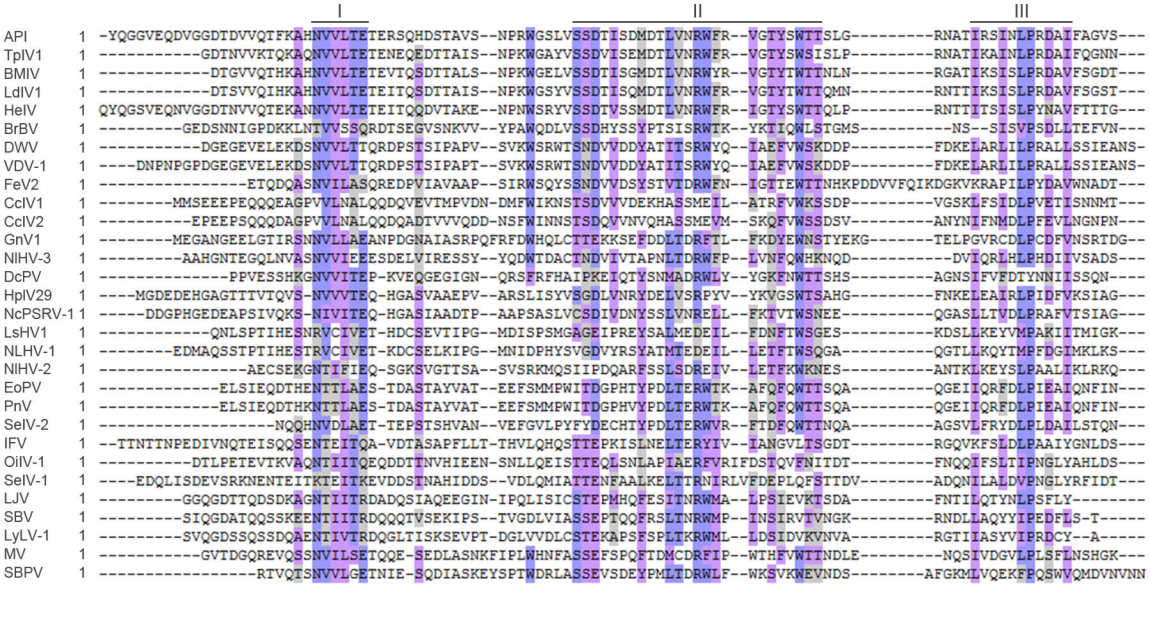
I
II
III
API 1 -YQGGVEQDVGGDTDVVQTFKAHNVVLTETERSQHDSTAVS--NPRWGSLVSSDTISDMDTLVNRWFR--VGTYSWTTSLG---------RNATIRSINLPRDAIFAGVS---
TpIV1 1 -----------GDTNVVKTQKAQNVVLTETENEQEDTTAIS--NPKWGAYVSSDVISEMDTLVNRWFR--VGTYSWSISLP---------RNATIKAINLPRDAIFQGNN---
BMIV 1 -----------DTGVVQTHKAHNVVLTETEVTQSDTTALS--NPKWGELVSSDTISGMDTLVNRWYR--VGTYTWTTNLN---------RGATIKSISLPRDAVFSGDT---
LdIV1 1 -----------DTSVVQIHKAHNVVLTETEITQSDTTAIS--NPKWGSYVSSDTISQMDTLVNRWFR--VGTYTWTTQMN---------RNTTIKSISLPRDAVFSGST---
HeIV 1 QYQGSVEQNVGGDTNVVQTEKANNVVLTETEITQQDVTAKE--NPNWSRYVSSDTVSSMDTLVNRWFR--IGTYSWTTQLP---------RNTTITSISLPYNAVFTTTG---
BrBV 1 ---------GEDSNNIGPDKKLNTVVSSQRDTSEGVSNKVV--YPAWQDLVSSDHYSSYPTSISRWTK--YKTIQWLSTGMS----------NS--SISVPSDLLTEFVN---
DWV 1 -----------DGEGEVELEKDSNVVLTTQRDPSTSIPAPV--SVKWSRWTSNDVVDDYATITSRWYQ--IAEFVWSKDDP---------FDKELARLILPRALLSSIEANS-
VDV-1 1 ----DNPNPGPDGEGEVELEKDSNVVLTTQRDPSTSIPAPT--SVKWSRWTSNDVVDDYATITSRWYQ--IAEFVWSKDDP---------FDKELARLILPRALLSSIEANS-
FeV2 1 ----------------ETQDQASNVILASQREDPVIAVAAP--SIRWSQYSSNDVVDSYSTVTDRWFN--IGTTEWTTNHKPDDVVFQIKDGKVKRAPILPYDAVWNADT---
CcIV1 1 --------MMSEEEPEQQQEAGPVVLNALQQDQVEVTMPVDN-DMFWIKNSTSDVVVDEKHASSMEIL--ATRFVWKSSDP---------VGSKLFSIDLPVETISNNMT---
CcIV2 1 ----------EPEEPSQQQDAGPVVLNALQQDQADTVVVQDD-NSFWINNSTSDQVVNVQHASSMEVM--SKQFVWSSDSV---------ANYNIFNMDLPFEVLNGNPN---
GnV1 1 --------MEGANGEELGTIRSNNVLLAEANPDGNAIASRPQFRFDWHQLCTTEKKSEFDDLTDRFTL--FKDYEWNSTYEKG-------TELPGVRCDLPCDFVNSRTDG--
NlHV-3 1 ---------AAHGNTEGQLNVASNVVIEEESDELVIRESSY--YQDWTDACTNDVIVTAPNLTDRWFP--LVNFQWHKNQD----------DVIQRLHLPHDIIVSADS---
DcPV 1 --------------PPVESSHKGNVVITEP-KVEQGEGIGN--QRSFRFHAIPKEIQTYSNMADRWLY--YGKFNWTTSHS---------AGNSIFVFDTYNNIISSQN----
HpIV29 1 ----MGDEDEHGAGTTIVTQVS-NVVVTEQ-HGASVAAEPV--ARSLISYVSGDLVNRYDELVSRPYV--YKVGSWTSAHG---------FNKELEAIRLPIDFVKSIAG---
NcPSRV-1 1 -----DDGPHGEDEAPSIVQKS-NIVITEQ-HGASIAADTP--AAPSASLVCSDIVDNYSSLVNRELL--FKTVTWSNEE----------QGASLLTVDLPRAFVTSIAG---
LsHV1 1 ------------QNLSPTIHESNRVCIVET-HDCSEVTIPG--MDISPSMGAGEIPREYSALMEDEIL--FDNFTWSGES----------KDSLLKEYVMPAKIITMIGK---
NLHV-1 1 --------EDMAQSSTPTIHESTRVCIVET-KDCSELKIPG--MNIDPHYSVGDVYRSYATMTEDEIL--LETFTWSQGA----------QGTLLKQYTMPFDGIMKLKS---
NlHV-2 1 ----------------AECSEKGNTIFIEQ-SGKSVGTTSA--SVSRKMQSIIPDQARFSSLSDREIV--LETFKWKNES----------ANTKLKEYSLPAALIKLRKQ---
EoPV 1 -------------ELSIEQDTHENTTLAES-TDASTAYVAT--EEFSMMPWITDGPHTYPDLTERWTK--AFQFQWTTSQA---------QGEIIQRFDLPIEAIQNFIN---
PnV 1 -------------ELSIEQDTHKNTTLAES-TDASTAYVAT--EEFSMMPWITDGPHVYPDLTERWTK--AFQFQWTTSQA---------QGEIIQRFDLPIEAIQNFIN---
SeIV-2 1 --------------------NQQHNVDLAET-TEPSTSHVAN--VEFGVLPYFYDECHTYPDLTERWVR--FTDFQWTTNQA---------AGSVLFRYDLPLDAILSTQN---
IFV 1 --TTNTTNPEDIVNQTEISQQSENTEITQA-VDTASAPFLLT-THVLQHQSTTEPKISLNELTERYIV--IANGVLTSGDT---------RGQVKFSLDLPAAIYGNLDS---
OiIV-1 1 ----------DTLPETEVTKVAQNTIITQEQDDTTNVHIEEN-SNLLQEISTTEQLSNLAPIAERFVRIFDSTQVFNITDT---------FNQQIFSLTIPNGLYAHLDS---
SeIV-1 1 ----EDQLISDEVSRKNENTEITKTEITKEVDDSTNAHIDDS--VDLQMIATTENFAALKELTTRNIRLVFDEPLQFSTTDV--------ADQNILALDVPNGLYRFIDT---
LJV 1 ---------GGQGDTTQDSDKAGNTIITRDADQSIAQEEGIN-IPQLISICSTEPMHQFESITNRWMA--LPSIEVKTSDA---------FNTILQTYNLPSFLY---------
SBV 1 ---------SIQGDATQQSSKEENTIITRDQQQTVSEKIPS--TVGDLVIASSEPTQQFRSLTNRWMP--INSIRVTVNGK---------RNDLLAQYYIPEDFLS-T-----
LyLV-1 1 ---------SVQGDSSQSSDQAENTIVTRDQGLTISKSEVPT-DGLVVDLCSTEKAPSFSPLTKRWML--LDSIDVKVNVA---------RGTIIASYVIPRDCY--A-----
MV 1 -----------GVTDGQREVQSSNVILSETQQE-SEDLASNKFIPLWHNFASSEFSPQFTDMCDRFIP--WTHFVWTTNDLE---------NQSIVDGVLPLSFLNSHGK---
SBPV 1 ----------------RTVQTSNVVLGETNIE-SQDIASKEYSPTWDRLASSEVSDEYPMLTDRWLF--WKSVKWEVNDS--------AFGKMLVQEKFPQSWVQMDVNVNN

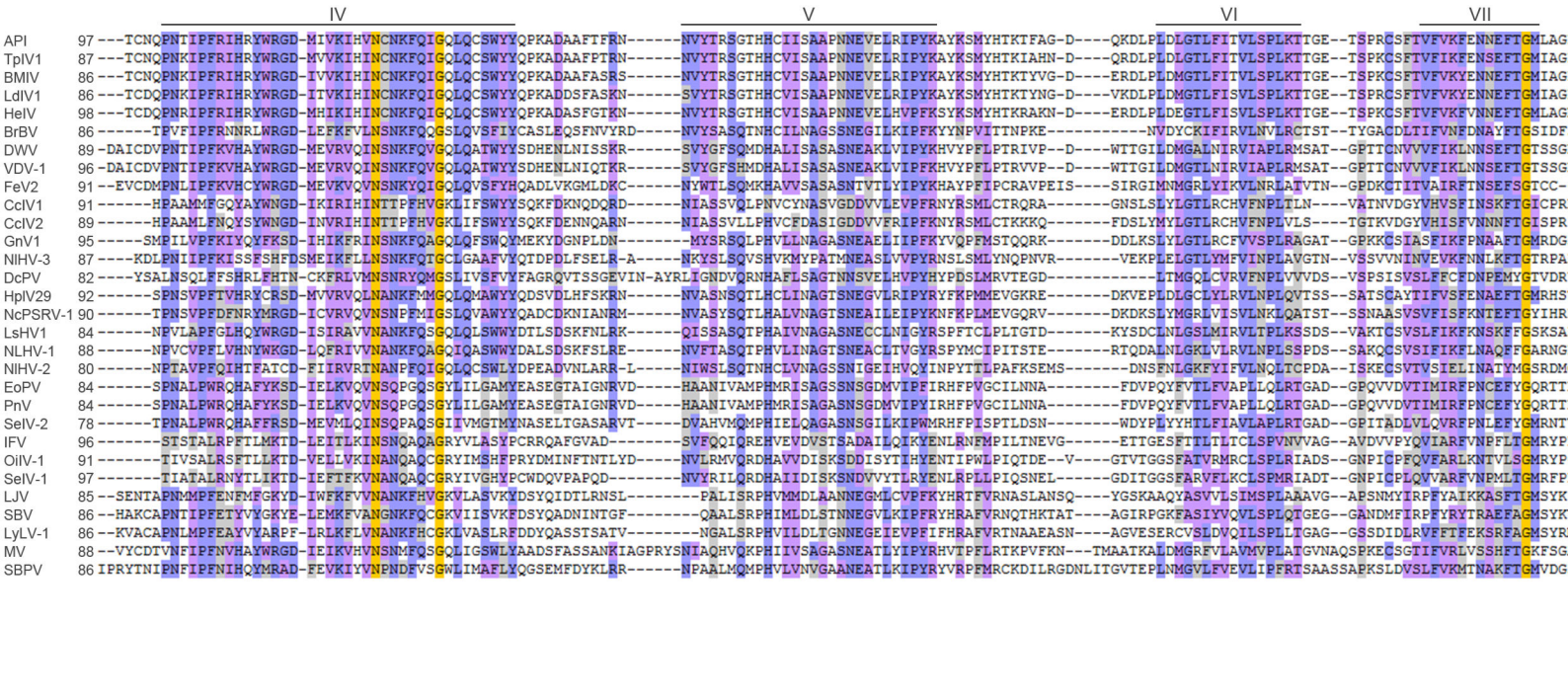
IV
V
VI
VII
API 97 ---TCNQPNTIPFRIHRYWRGD-MIVKIHVNCNKFQIGQLQCSWYYQPKADAAFTFRN------NVYTRSGTHHCIISAAPNNEVELRIPYKAYKSMYHTKTFAG-D----QKDLPLDLGTLFITVLSPLKTTGE--TSPRCSFTVFVKFENNEFTGMLAG
TpIV1 87 ---TCNQPNKIPFRIHRYWRGD-MVVKIHINCNKFQIGQLQCSWYYQPKADAAFPTRN------NVYTRSGTHHCVISAAPNNEVELRIPYKAYKSMYHTKIAHN-D----QRDLPLDLGTLFITVLSPLKTTGE--TSPKCSFTVFIKFENSEFTGMIAG
BMIV 86 ---TCNQPNKIPFRIHRYWRGD-IVVKIHINCNKFQIGQLQCSWYYQPKADAAFASRS------NVYTRSGTHHCVISAAPNNEVELRIPYKAYKSMYHTKTYVG-D----ERDLPLDMGTLFITVLSPLKTTGE--TSPKCSFTVFVKYENNEFTGMIAG
LdIV1 86 ---TCDQPNKIPFRIHRYWRGD-ITVKIHINCNKFQIGQLQCSWYYQPKADDSFASKN------SVYTRSGTHHCVISAAPNNEVELRIPYKAYKSMYHTKTYNG-D----VKDLPLDMGTLFISVLSPLKTTGE--TSPRCSFTVFVKYENNEFTGMIAG
HeIV 98 ---TCDQPNRIPFRIHRYWRGD-MHIKIHINCNKFQIGQLQCSWYYQPKADASFGTKN------NVYTRSGTHHCVISAAPNNEVELHVPFKSYKSMYHTKRAKN-D----ERDLPLDEGTLFISVLSPLKTTGE--TSPKCSFTVFVKFVNNEFTGMLAG
BrBV 86 ------TFVFIPFRNNRLWRGD-LEFKFVLNSNKFQQGSLQVSFIYCASLEQSFNVYRD------NVYSASQTNHCILNAGSSNEGILKIPFKYYNPVITTNPKE----------NVDYCKIFIRVLNVLRCTST--TYGACDLTIFVNFDNAYFTGSIDF
DWV 89 -DAICDVPNTIPFKVHAYWRGD-MEVRVQINSNKFQVGQLQATWYYSDHENLNISSKR------SVYGFSQMDHALISASASNEAKLVIPYKHVYPFLPTRIVP--D----WTTGILDMGALNIRVIAPLRMSAT--GPTTCNVVVFIKLNNSEFTGTSSG
VDV-1 96 -DAICDVPNTIPFKVHAYWRGD-MEVRVQINSNKFQVGQLQATWYYSDHENLNIQTKR------SVYGFSHMDHALISASASNEAKLVIPFKHVYPFLPTRVVP--D----WTTGILDMGTLNIRVIAPLRMSAT--GPTTCNVVVFIKLNNSEFTGTSSG
FeV2 91 --EVCDMPNLIPFKVHCYWRGD-MEVKVQVNSNKYQIGQLQVSFYHQADLVKGMLDKC------NYWTLSQMKHAVVSASASNTVTLYIPYKHAYPFIPCRAVPEIS-----SIRGIMNMGRLYIKVLNRLATVTN--GPDKCTITVAIRFTNSEFSGTCC-
CcIV1 91 ------HPAAMMFGQYAYWNGD-IKIRIHINTTPFHVGKLIFSWYYSQKFDKNQDQRD------NIASSVQLPNVCYNASVGDDVVLEVPFRNYRSMLCTRQRA-------GNSLSLYLGTLRCHVFNPLILN----VATNVDGYVHVSFINSKFTGICPR
CcIV2 89 ------HPAAMLFNQYSYWNGD-INVRIHINTTPFHVGKLIFSWYYSQKFDENNQARN------NIASSVLLPHVCFDASIGDDVVFRIPFKNYRSMLCTKKKQ-------FDSLYMYLGTLRCHVFNPLVLS----TGTKVDGYVHISFVNNNFTGISPR
GnV1 95 -----SMPILVPFKIYQYFKSD-IHIKFRINSNKFQAGQLQFSWQYMEKYDGNPLDN--------MYSRSQLPHVLLNAGASNEAELIIPFKYVQPFMSTQQRK-------DDLKSLYLGTLRCFVVSPLRAGAT--GPKKCSIASFIKFPNAAFTGMRDG
NlHV-3 87 ----KDLPNIIPFKISSFSHFDSMEIKFLLNSNKFQTGCLGAAFVYQTDPDLFSELR-A-----NKYSLSQVSHVKMYPATMNEASLVVPYRNSLSMLYNQPNVR-------VEKPLELGTLYMFVINPLAVGTN--VSSVVNINVEVKFNNLKFTGTRPA
DcPV 82 ----YSALNSQLFFSHRLFHTN-CKFRLVMNSNRYQMGSLIVSFVYFAGRQVTSSGEVIN-AYRLIGNDVQRNHAFLSAGTNNSVELHVPYHYPDSLMRVTEGD-----------LTMGQLCVRVFNPLVVVDS--VSPSISVSLFFCFDNPEMYGTVDR
HpIV29 92 ------SPNSVPFTVHRYCRSD-MVVRVQLNANKFMMGQLQMAWYYQDSVDLHFSKRN------NVASNSQTLHCLINAGTSNEGVLRIPYRYFKPMMEVGKRE-------DKVEPLDLGCLYLRVLNPLQVTSS--SATSCAYTIFVSFENAEFTGMRHS
NcPSRV-1 90 ------TPNSVPFDFNRYMRGD-ICVRVQVNSNPFMIGSLQVAWYYQADCDKNIANRM------NVASYSQTLHALVNAGTSNEAILEIPYKNFKPLMEVGQRV-------DKDKSLYMGRLVISVLNKLQATST--SSNAASVSVFISFKNTEFTGYIHR
LsHV1 84 ------NPVLAPFGLHQYWRGD-ISIRAVVNANKFQSGQLQLSWWYDTLSDSKFNLRK------QISSASQTPHAIVNAGASNECCLNIGYRSPFTCLPLTGTD-------KYSDCLNLGSLMIRVLTPLKSSDS--VAKTCSVSLFIKFKNSKFFGSKSA
NLHV-1 88 ------NPVCVPFLVHNYWKGD-LQFRIVVNANKFQAGQIQASWWYDALSDSKFSLRE------NVFTASQTPHVLINAGTSNEACLTVGYRSPYMCIPITSTE-------RTQDALNLGKLVLRVLNPLSSPDS--SAKQCSVSIFIKFLNAQFFGARNG
NlHV-2 80 ------NPTAVPFQIHTFATCD-FIIRVRINANPFQIGQLQCSWLYDPEADVNLARR-L-----NIWSLSQTNHCLVNAGSSNIGEIHVQYINPYTTLPAFKSEMS-------DNSFNLGKFYIFVLNQLTCPDA--ISKECSVTVSIELINATYMGSRDM
EoPV 84 ------SPNALPWRQHAFYKSD-IELKVQVNSQPGQSGYLILGAMYEASEGTAIGNRVD-----HAANIVAMPHMRISAGSNSGDMVIPFIRHFPVGCILNNA--------FDVPQYFVTLFVAPLLQLRTGAD--GPQVVDVTIMIRFPNCEFYGQRTT
PnV 84 ------SPNALPWRQHAFYKSD-IELKVQVNSQPGQSGYLILGAMYEASEGTAIGNRVD-----HAANIVAMPHMRISAGASNSGDMVIPYIRHFPVGCILNNA--------FDVPQYFVTLFVAPLLQLRTGAD--GPQVVDVTIMIRFPNCEFYGQRTT
SeIV-2 78 ------TPNALPWRQHAFFRSD-MEVMLQINSQPAQSGIIVMGTMYNASELTGASARVT-----DVAHVMQMPHIELQAGASNSGILKIPWMRHFPISPTLDSN--------WDYPLYYHTLFIAVLAPLRTGAD--GPITADLVLQVRFPNLEFYGMRNT
IFV 96 -------STSTALRPFTLMKTD-LEITLKINSNQAQAGRYVLASYPCRRQAFGVAD--------SVFQQIQREHVEVDVSTSADAILQIKYENLRNFMPILTNEVG------ETTGESFTTLTLTLCLSPVNVVAG--AVDVVPYQVIARFVNPFLTGMRYP
OiIV-1 91 -------IIVSALRSFTLLKTD-VELLVKINANQAQCGRYIMSHFPRYDMINFTNTLYD-----NVLRMVQRDHAVVDISKSDDISYTIHYENTIPWLPIQTDE--V----GTVTGGSFATVRMRCLSPLRIADS--GNPICPFQVFARLKNTVLSGMRYP
SeIV-1 97 -------TIATALRNYTLIKTD-IEFTFKVNANQAQCGRYIVGHYPCWDQVPAPQD--------NVYRILQRDHAIIDISKSNDVVYTLRYENLRPLLPIQSNEL------GDITGGSFARVFLKCLSPMRIADT--GNPICPLQVVARFVNPMLTGMRFP
LJV 85 --SENTAPNMPFENFMFGKYD-IWFKFVVNANKFHVGKVLASVKYDSYQIDTLRNSL--------PALISRPHVMMDLAANNEGMLCVPFKYHRTFVRNASLANSQ----YGSKAAQYASVVLSIMSPLAAAVG--APSNMYIRPFYAIKKASFTGMSYK
SBV 86 --HAKCAPNTIPFETYVYGKYE-LEMKFVANGNKFQCGKVIISVKFDSYQADNINTGF--------QAALSRPHIMLDLSTNNEGVLKIPFRYHRAFVRNQTHKTAT----AGIRPGKFASIYVQVLSPLQTGEG--GANDMFIRPFYRYTRAEFAGMSYK
LyLV-1 86 --KVACAPNLMPFEAYVYARPF-LRLKFLVNANKFHCGKLVASLRFDDYQASSTSATV--------NGALSRPHVILDLTGNNEGEIEVPFIFHRAFVRTNAAEASN----AGVESERCVSLDVQILSPLLTGAG--GSSDIDLRVFFTFEKSRFAGMSYR
MV 88 --VYCDTVNFIPFNVHAYWRGD-IEIKVHVNSNMFQSGQLIGSWLYAADSFASSANKIAGPRYSNIAQHVQKPHIIVSAGASNEATLYIPYRHVTPFLRTKPVFKN---TMAATKALDMGRFVLAVMVPLATGVNAQSPKECSGTIFVRLVSSHFTGKFSG
SBPV 86 IPRYTNIPNFIPFNIHQYMRAD-FEVKIYVNPNDFVSGWLIMAFLYQGSEMFDYKLRR------NPAALMQMPHVLVNVGAANEATLKIPYRYVRPFMRCKDILRGDNLITGVTEPLNMGVLFVEVLIPFRTSAASSAPKSLDVSLFVKMTNAKFTGMVDG

B VP3

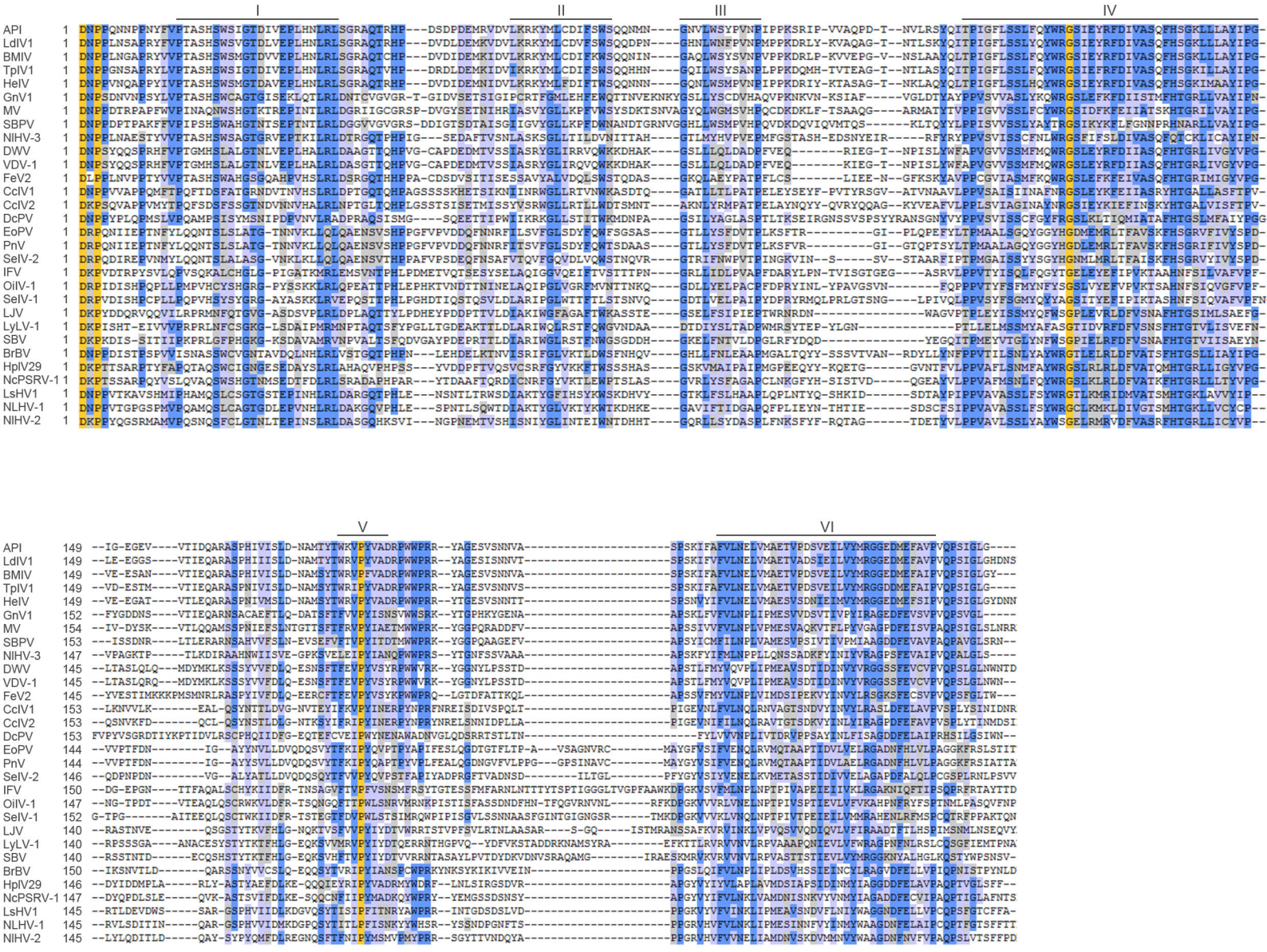
I
II
III
IV
V
VI
API
LdIV1
BMIV
TpIV1
HeIV
GnV1
MV
SBPV
NIHV-3
DWV
VDV-1
FeV2
CcIV1
CcIV2
DcPV
EoPV
PnV
SeIV-2
IFV
OiIV-1
SeIV-1
LJV
LyLV-1
SBV
BrBV
HpIV29
NcPSRV-1
LsHV1
NLHV-1
NIHV-2

C VP1

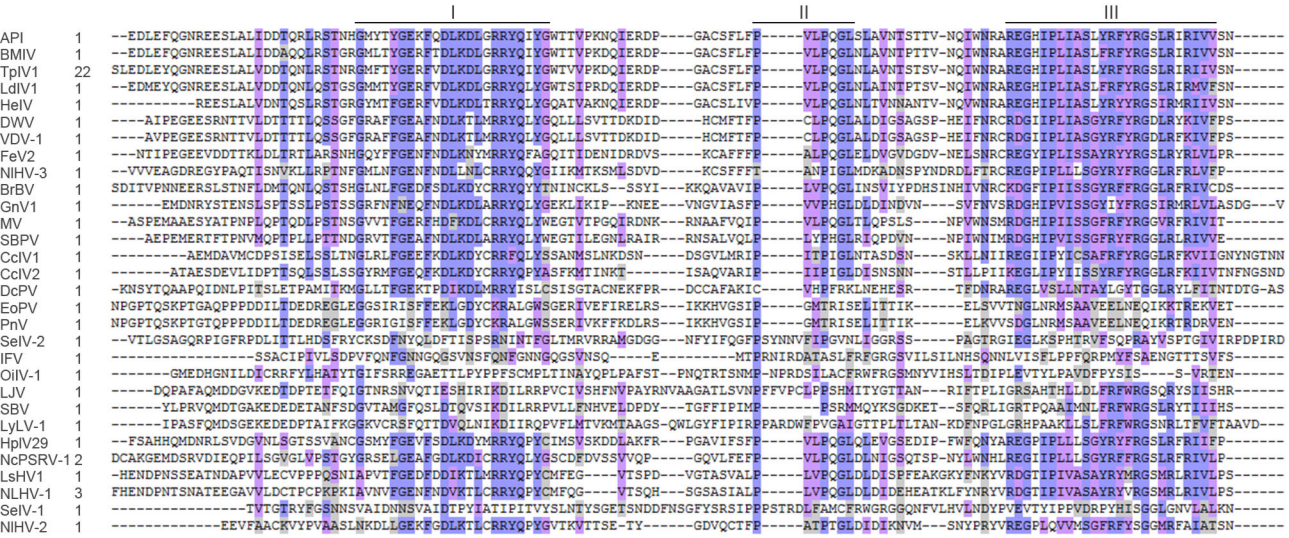
I
II
III
API 1 --EDLEFQGNREESLALIDDTQRLRSTNHGMYTYGEKFQDLKDLGRRYQIYGWTTVPKNQIERDP----GACSFLFP-----VLPQGLSLAVNTSTTV-NQIWNRAREGHIPLIASLYRFYRGSLRIRIVVSN------
BMIV 1 --EDLEFQGNREESLALIDDAQQLRSTGRGMLTYGERFTDLKDLGRRYQIYGWTTVPKDQIERDP----GACSFLFP-----VLPQGLNLAVNTPTTV-NQIWNRAREGHIPLIASLFRFYRGSLRIRIVVSN------
TplV1 22 SLEDLEYQGNREESLALVDDTQNLRSTNRGMFTYGERFVDLKDLGRRYQIYGWTVVPKDQIERDP----GACSFLFP-----VLPQGLNLAVNTSTSV-NQIWNRAREGHIPLIASLYRFYRGSLRIRIIVSN------
LdlV1 1 --EDMEYQGNREESLALVDDTQNLQSTGSGMMTYGERFVDLKDLGRRYQLYGWTSIPRDQIERDP----GACSFLFP-----VLPQGLNLAINTPTSV-NQIWNRAREGHIPLIASLFRFYRGSLRIRMVFSN------
HelV 1 ----------REESLALVDNTQSLRSTGRGYMTFGERFVDLKDLTRRYQLYGQATVAKNQIERDP----GACSLIVP-----VLPQGLNLIVNNANTV-NQVWNRAREGHIPLIASLYRYYRGSIRMRIIVSN------
DWV 1 ----AIPEGEESRNTTVLDTTTTLQSSGFGRAFFGEAFNDLKTLMRRYQLYGQLLLSVTTDKDID-----HCMFTFP-----CLPQGLALDIGSAGSP-HEIFNRCRDGIIPLIASGYRFYRGDLRYKIVFPS------
VDV-1 1 ----AVPEGEESRNTTVLDTTTTLQSSGFGRAFFGEAFNDLKTLMRRYQLYGQLLLSVTTDKDID-----HCMFTFP-----CLPQGLALDIGSAGSP-HEIFNRCRDGIIPLIASGYRFYRGDLRFKIVFPS------
FeV2 1 ---NTIPEGEEVDDTTKLDLTRTLARSNHGQYFFGENFNDLKNYMRRYQFAGQITIDENIRDVS-----KCAFFFP-----ALPQGLELDVGVDGDV-NELSNRCREGYIPLISSAYRYYRGSLRYRLVLPR------
NIHV-3 1 --VVVEAGDREGYPAQTISNVKLLRPTNFGMLNFGENFNDLLNLCRRYQQYGIIKMTKSMLSDVD-----KCSFFFT-----ANPIGLMDKADNSPYNDRDLFTRCREGPIPLLLSGYRYFRGGLRFRLVFP-------
BrBV 1 SDITVPNNEERSLSTNFLDMTQNLQSTSHGLNLFGEDFSDLKDYCRRYQYTNINCKLS--SSYI---KKQAVAVIP-----LVPQGLINSVIYPDHSINHIVNRCKDGFIPIISSGYRFFRGGLRFRIVCDS------
GnV1 1 ------EMDNRYSTENSLSPTSSLPSTSSGRFNFNEQFNDLKDLARRYQLYGEKLIKIP--KNEE---VNGVIASFP-----VVPHGLDLDINDVN----SVFNVSRDGHIPVISSGYIYFRGSIRMRLVLASDG---V
MV 1 --ASPEMAAESYAIPNPLQPTQDLPSTNSGVVTFGERFHDFKDLCRRYQLYWEGTVTPGQIRDNK---RNAAFVQIP-----VLPQGLTLQPSLS----NPVWNSMRDGHIPIISSGFRFYRGGVRFRIVIT-------
SBPV 1 ----AEPEMERTFTPNVMQPTPLLPTTNDGRVTFGEAFNDLKDLARRYQLYWEGTILEGNLRAIR---RNSALVQLP-----LYPHGLRIQPDVN----NPIWNIMRDGHIPVISSGFRYFRGGLRLRIVVE-------
CclV1 1 ---------AEMDAVMCDPSISELSSLTNGLRLFGEEFKDLKDYCRRFQLYSSANMSLNKDSN-----DSGVLMRIP-----ITPIGLNTASDSN----SKLLNIIREGIIPYICSAFRFYRGGLRFKVIIGNYNGTNN
CclV2 1 -------ATAESDEVLIDPTTSQLSSLSSGYRMFGEQFKDLKDYCRRYQPYASFKMTINKT-------ISAQVARIP-----IIPIGLDISNSNN----STLLPIIKEGLIPYIISSYRFYRGGLRFKIIVTNFNGSND
DcPV 1 -KNSYTQAAPQIDNLPITSLETPAMITKMGLLTFGEKTPDIKDLMRRYISLCSISGTACNEKFPR---DCCAFAKIC-----VHPFRKLNEHESR-----TFDNRAREGLVSLLNTAYLGYTGGLRYLFITNTDTG-AS
EoPV 1 NPGPTQSKPTGAQPPPDDILTDEDREGLEGGSIRISFFEKLGDYCKRALGWSGERIVEFIRELRS---IKKHVGSIP-----GMTRISELITTIK------ELSVVTNGLNRMSAAVEELNEQIKKTREKVET------
PnV 1 NPGPTQSKPTGTQPPPDDILTDEDREGLEGGRIGISFFEKLGDYCKRALGWSSERIVKFFKDLRS---IKKHVGSIP-----GMTRISELITTIK------ELKVVSDGLNRMSAAVEELNEQIKKTRDRVEN------
SelV-2 1 --VTLGSAGQRPIGFRPDLITTLHDSFRYCKSDFNYQLDFTISPSRNINITFGLTMRVRRAMGDGG---NFYIFQGFFSYNNVFIPGVNLIGGRSS-----PAGTRGIEGLKSPHTRVFSQPRAYVSPTGIVIRPDPIRD
IFV 1 ----------------SSACIPIVLSDPVFQNFGNNGQGSVNSFQNFGNNGQGSVNSQ-----E---------MTPRNIRDATASLFRFGRGSVILSILNHSQNNLVISFLPPFQRPMYFSAENGTTTSVFS------
OilV-1 1 -------GMEDHGNILDICRRFYLHATYTGIFSRREGAETTLPYPPFSCMPLTINAYQPLPAFST--PNQTRTSNMP-NPRDSILACFRWFRGSMNYVIHSLTDIPLEVTYLPAVDFPYSIS----S-VRTEN------
LJV 1 -----DQPAFAQMDDGVKEDTDPTETFQIGTNRSNVQTIESHIRIKDILRRPVCIVSHFNVPAYRNVAAGATLSVNPFFVPCLPFSHMITYGTTAN---RIFTPLIGRSAHTHLLDLFRFWRGSQRYSIISHR------
SBV 1 ------YLPRVQMDTGAKEDEDETANFSDGVTAMGFQSLDTQVSIKDILRRPVLLFNHVELDPDY---TGFFIPIMP-------FSRMMQYKSGDKET--SFQRLIGRTPQAAIMNLFRFWRGSLRYTIIIHS------
LyLV-1 1 ------IPASFQMDSGEKEDEDPTAIFKGGKVCRSFQTTDVQLNIKDIIRQPVFLMTVKMTAAGS-QWLGYFIPIRPPARDWFPVGAIGTTPLTLTAN-KDFNPGLGRHPAAKLLSLFRFWRGSNRLTFVFTAAVD---
HplV29 1 --FSAHHQMDNRLSVDGVNLSGTSSVANCGSMYFGEVFSDLKDYMRRYQPYCIMSVSKDDLAKFR---PGAVIFSFP-----VLPQGLQLEVGSEDIP-FWFQNYARERGPIPLLLSGYRYFRGSLRFRIIFP------
NcPSRV-1 2 DCAKGEMDSRVDIEQPILSGVGLVPSTGYGRSELGEAFGDLKDICRRYQLYGSCDFDVSSVVQP-----GQVLFEFP-----VLPQGLDLNIGSQTSP-NYLWNHLREGIIPLLLSGYRFFRGSLRFRIVLP------
LsHV1 1 -HENDPNSSEATNDAPVVLECVPPPQSNIAPVTFGEDFDDIKTLMRRYQPYCMFEG----VTSPD---VGTASVALP-----LVPQGLDLDISPFEAKGKYFNKYVRDGTIPIVASAYRYMRGSMRLRIVLPS------
NLHV-1 3 FHENDPNTSNATEEGAVVLDCTPCPKPKIAVNVFGENFNDVKTLCRRYQPYCMFQG----VTSQH---SGSASIALP-----LVPQGLDLDIDEHEATKLFYNRYVRDGTIPIVASAYRYVRGSMRLRIVLPS------
SelV-1 1 ----------------TVTGTRYFGSNNSVAIDNNSVAIDTPYIATIPITVYSLNTYSGETSNDDFNSGFYSRSIPPPSTRDLFAMCFRWGRGGQNFVLHVLNDYPVEVTYIPPVDRPYHISGGLGNVLALKN------
NIHV-2 1 ------------EEVFAACKVYPVAASLNKDLLGEKFGDLKTLCRRYQPYGVTKVTTSE-TY------GDVQCTFP-----ATPTGLDIDIKNVM---SNYPRYVREGPLQVVMSGFRFYSGGMRFAIATSN------
IV
V
VI
VII
API 122 --APDLIWWVQHRPDRR---LDRDVITPCTQVSTAEAVFNHTYGVYMQALRVNNVIEIEVPYYQMANYGLLQKPIISE--GPTVQDWSRFYSLGELSVGFF------GEQPSAD--VRCTIYYSMADDCRFTTYQGVPPMVLLDDLPE
BMIV 122 --APDLVMWVQHRPDRR---LDRDVITPCTQVSTAEAVFNHTYGVYMQALSVNNVVEIEVPFYQMANFGLLQKPVLSE--GKPVQDWSRFYSLGELSVGFF------GDQPSTD--VRCTIYYSMADDCRFTTYQGVPPMVLIDDLPE
TplV1 145 --ASDLVMWVQHRPDRR---LDRDVITPCTQVTTAEAVFNHSYGVYMQALNVNNVVEIEVPFYQMANFGLLQKPVISE--N--VSDWSRFYSLGELSIGFF------GEKPDND--VRCTIYYSMADDCRFSTYQGVPPMVLLDDLPE
LdlV1 122 --ASGLTAWVQHRPDRR---LDRDVITPCTQVTTAEAVFNHSYGVYMQSLSVNNMIEIEVPFYQMANFGLLQKPIIAV--GGPVTDWSRFYSLGELSVGFF------GDKPTDD--IRCTIYYSMADDCRFSTYQGVPPVVLIDDLPE
HelV 114 --GIGLTMWVQHRPDRR---LSRNTIIPCTAVSTAEAVFNHTYGMYMQDLNVNNVIEIEVPFYQMANFGLLQQPVVTE--GPPYKDWTKFYSLGELSIGFF------GDQPSDD--IRVTIFYALADDCRFTTYQGVPPMVIIDDLPE
DWV 119 --NVNSNIWVQHRPDRRLEGWSAAKIVNCDAVSTGQGVYNHGYASHIQITRVNNVIELEVPFYNATCYNYLQAFNA------SSAASSYAVSLGEISVGFQA----TSDDIASIVNKPVTIYYSIGDGMQFSQWVGYQPMMILDQLPA
VDV-1 119 --NVNSNIWVQHRPDRRLKGWSEAKIVNCDAVSTGQGVYNHGYASHIQITRVNNVIELEVPFYNATCYNYLQAFNP------SSAASSYAVSLGEISVGFQA----TSDDIAAIVNKPVTIYYSIGDGMQFSQWVGYQPMMILDQLPA
FeV2 120 --EIDMLVWIQHRPDR---VYTRRKVQLCTSVETSQSVYNHGYGSYIQFAKVNNVVEFEIPFYNDSCYNWLQNPVDN-----LDNGHKLAITLGEVAVGLQL----SHDDLAKIKGKTISIYYALADDMTFTHWVGFPLLY-------
NIHV-3 121 -ACKDTLIWIQHRSDRP---SNPMTLQACTTVESAQALMNHGYATEIQLANINSIVEFEVPFYQYDAYGLLQVPPYP---SSAGVAYDYQFGLGQVMVGIRC----SKEDSALFTDRYCEIFYSLADDFSPSLFYGFPPTIML-----
BrBV 124 --RRNVNLWVQHRPEMLLK--YLQPHMTDIDNQSLSDYFNHSYSFNIQNLSLNNVVSLEVPWYSKNMLGCSFFPKVISSDTDINSQNLTSFSLGSLILGLD------YDKTETTNPLNLDIYYSLADDFHFNTFQGFPLMLTLEQIPN
GnV1 116 VTLGGVKIWVPHHPDGDCR---THALEVYPGIKSQDAFRSHGYSFYMQAMNVNSIVEFEVPFYQSGMYGLTRKMSSHA--K--NSKVCQYYSLGNIVIGAS------AGKTDKAVDFRLQVYYSIGDDFSFNVFRGFPRTVF------
MV 119 --GLDDSVWVQHHPDRQ---FIGQVPILGKDIHDKDAYRNHTYGFHVQNLSVNRTIEVEIPFYKPGIYNLLG-----D--LALDYDCKQYGTLGDVVIGLE------GDQAVDS-AIDIAIYYTISDDCSFNVFCGFPDMVFCDEVYK
SBPV 117 --GLNSCVWVQHHPDRR---SIFSRPIIGRYIAAKDAYRNHAYAAYVQNMSVNRTIEVEVPFYQPGLYGMLNASDN-N--TANSFDRLRFTGLGDLLIGIE------GEQPIPKEGIEISVYYSIADDFSFNIFCGFPPMVYCDETYS
CclV1 116 NIGGQDTIYIQHKPDIIAD--TRDLSTNTINASTRTRLFQSGYAYTALSTSVNNSITVEVPCYVPTNLLMLQKPD------YNQTSEIMHYSLGVLDIYQQ------KPQINEIVNTNVTLHYSFADDMDLSCFIGFPPMIPLAQ---
CclV2 116 DKAGHTVFYIQHKPDIIVS--DRNIKVTTLSTNSKEALFQSGYAYSALSTSVNNSLTVEIPCYIPTNLLMLQKPN------FTALSEILHYTLGVLDIYLK------RPTATESGDYMIDIHYAFADDMDMSCFVGVPPVTSTTNPIL
DcPV 124 SFPEGTMIYVQHRYDDDIG-WDKALVESSSPVRELKDFMLTSYATFSQSLTVNNTFRVEVPWYQKEEFRYCVP----------CRAKFNNGRLYVWIH---------CPVDVKVNIEVYYSYSDDTDNICWQGFPELLNLTTITP
EoPV 120 --------FVGGVGGKLN---SLDTGDLVSNGIEYVAYILNIYNSK----------SVGMTLINVAALLSKMGLG-------RYLVNDLVDRLGTFAKGEDT----EEIEREYTSLIITGVLGAFGLGTMNVEKEGFVKPFLSNVKDF
PnV 120 --------FVGGVGGKLS---SLDMGDLVSNGIEYVAYILNIYNSRSVGMTLLNVRSVGMTLLNVAALLSKMGLG-------HYIVNDLVDRLGTFAKGDNT----DEIEREYTSLIITGVLGALGLGTMNVEKEGFVKPFLSNVKDF
SelV-2 129 DLTKDGDVESNPGFVFS----RPEAGPSSDPPGSHQPDPESEYFTTEEQEEMGRAGMTRRLYNKVSSYLRTVRRMPR---DTVDEFWRRYEEKKRIVVDLT-------DSVRTVSEFVKHVEDSNMDKQKFERAFEAFESIGKASENF
IFV 103 --------LGVARTSIG----LSAPDSQGYASVRNVPSVNPVTDIEIPFYSSAVYMDLQARNGLGAALAIMGNSQS---MARGPLYDVVTRTLGTLKIQTELYPFPATAENLARLQSTVTIRMGLSDDFKFHHFMGTPPVIANYPLVP
OilV-1 119 ----VDNVFHTSNTDIP---IFATPINIGLPTEVIEPRLNPILTVEVPMYNANNYIDVLGAFSGDAFWSQNVRNR-R----YNEINNLTSLYLGNLVIRGING-QVLPLNQAMNQNFKVRVLSAFGDDAQFHHFIGCPPLNR------
LJV 126 --VTGAPIYASYVPHSGAMNCGTVTFLTVDLLDTXCPPSSFGLATEIMIPSVNPTMSIEVPYETENNWTLMQCEN--------WERNFSWRDHGDYNVGHIV--------LWSEEAFTCDIFWSAGDDFDVKNFLGPPPVIAPFSKFA
SBV 116 --TDGHPIYVTHVPHTGN-RVYGLMKVNNLHEYTKVPIFGCGLTTEMIIPSVNPSICVEVPFDTENNWAVTFDED--------AQRNYSWRDKGDTVTGHL-------V-VTPVVSVYMSVWVEAGDDFEVSNFYGPPSVKTNDWNYA
LyLV-1 128 ---DRVPIYIVHLPHSG---AELYGSFATTSTDLPVGLHQTGLRTTMVIPGINNTEVVEFPYDTENNWSLMYRDN--------PASKFRIRDMGDYNAGHVV---------LLSAQGFNADVWWEAGDDFEFSNFYGPAIQYTNSNAGA
HplV29 122 --DSDAIWFVQHIPNGRWHDSLVWQRKTTDYSLGLEHMINHSYAYTVQQGSINRVISFEVPFYLPYDACILQRFDQN---KIRHTDLMSAATLGEIRVGLLS----LGALKDTEAYPVIQVAYSFGDDARPSTFQGFPPVLLNQDIPL
NcPSRV-1 123 --DTNTSYFVQHIPYAYR---KERKIKPIASYVDGTNAFNHGYAYTVQSRFLNNVLSFEVPYYLNVDCMMLQQSE-LPSSIKGATDLAFALSLGVVRIGSLK---KIPLNLKKKINFNFEIAYALGDDARPSVFQGFSPVIFTNDIPP
LsHV1 121 --DVKCNIWVQHRPDSH---ADKWLPKMLGEHDVTSCYLNTGYSVGFQTTKVNNTYSIEIPYYQPFSFILSQRTDLK------RPVAQSVSSLGTLFIGFE-------QNSGTVKRFPVSVFYSLGDDAFFEYSQGFPPMIPISSSTP
NLHV-1 124 --DVRCNVWTQHRADIH---MDKWSVKPIAESGVAACYLNTGYAVCFQTTKVNNTLSIEIPYYQPYAFVLNQRTDLK------RGGALQISSLGTLYIGFE-------QNTSTPQKFPVSVFYALGDDCFFEYFQGFPPMIPLARRAL
SelV-1 118 ----TNENFITSRNAYP---IYAMPSATGYPNEVIEPRVNPLLTIENPMMNINNYMDLQAPLWSFTDSIPNLNNYPQQDLVSLTNGQLLIRQLANVSTTDPS------VASVSTNAFRIRVQSALADDFMFYHFMGVPPTMLTSVNFW
NIHV-2 106 ---DQFSLWVQHRPDYE----GELKINTISNKKTASNLINSGYAQYLQSLKVNNIVTFEVPYYKNRNCLYAQRVK-------KLKRTANTATLGQVVLGIE------G--KKKDDKLAMTIFASVADDFQAYVFEQGFPPMIYVDDLQV

D Hel

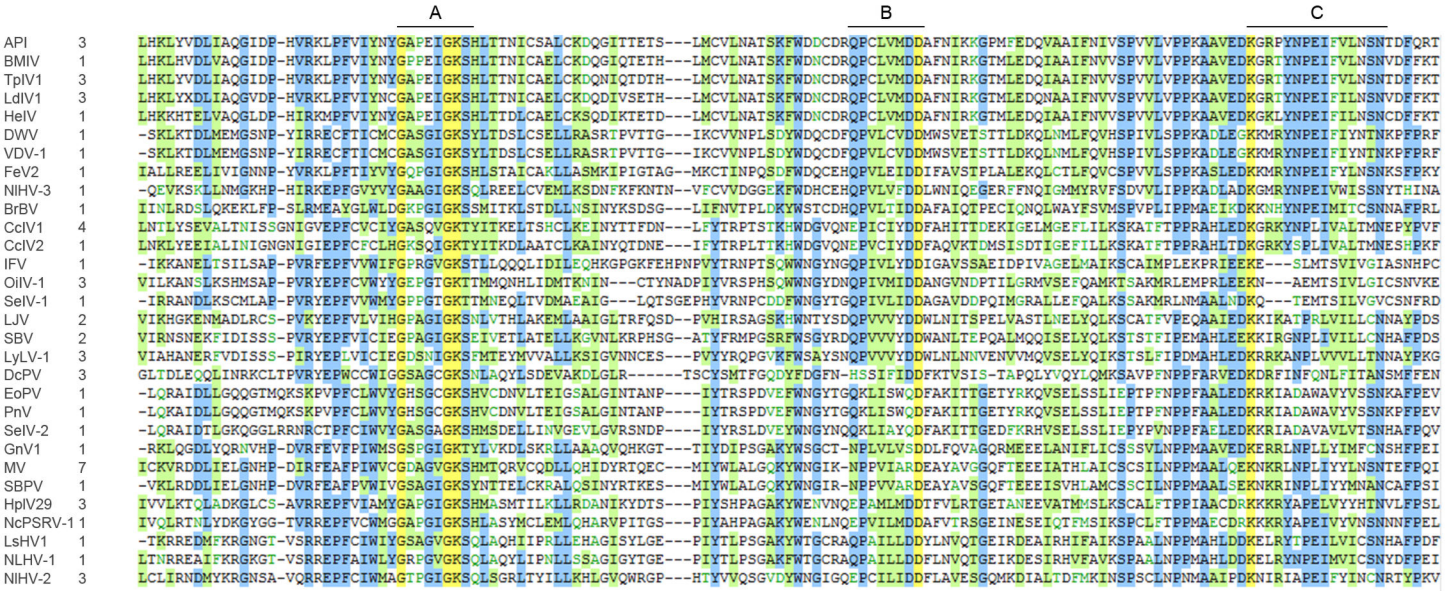
A
B
C
API 3
BMIV 1
TpIV1 3
LdIV1 3
HeIV 1
DWV 1
VDV-1 1
FeV2 1
NlHV-3 1
BrBV 1
CcIV1 4
CcIV2 1
IFV 1
OiIV-1 3
SeIV-1 1
LJV 2
SBV 2
LyLV-1 3
DcPV 3
EoPV 1
PnV 1
SeIV-2 1
GnV1 1
MV 7
SBPV 1
HpIV29 3
NcPSRV-1 1
LsHV1 1
NLHV-1 1
NlHV-2 3

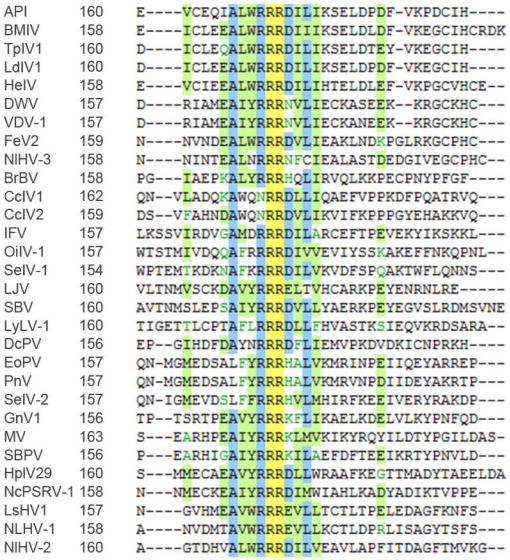
API 160
BMIV 158
TpIV1 160
LdIV1 160
HeIV 158
DWV 157
VDV-1 157
FeV2 159
NlHV-3 158
BrBV 158
CcIV1 162
CcIV2 159
IFV 157
OiIV-1 157
SeIV-1 154
LJV 160
SBV 160
LyLV-1 160
DcPV 156
EoPV 157
PnV 157
SeIV-2 157
GnV1 156
MV 163
SBPV 156
HpIV29 160
NcPSRV-1 158
LsHV1 157
NLHV-1 158
NlHV-2 160

E Pro

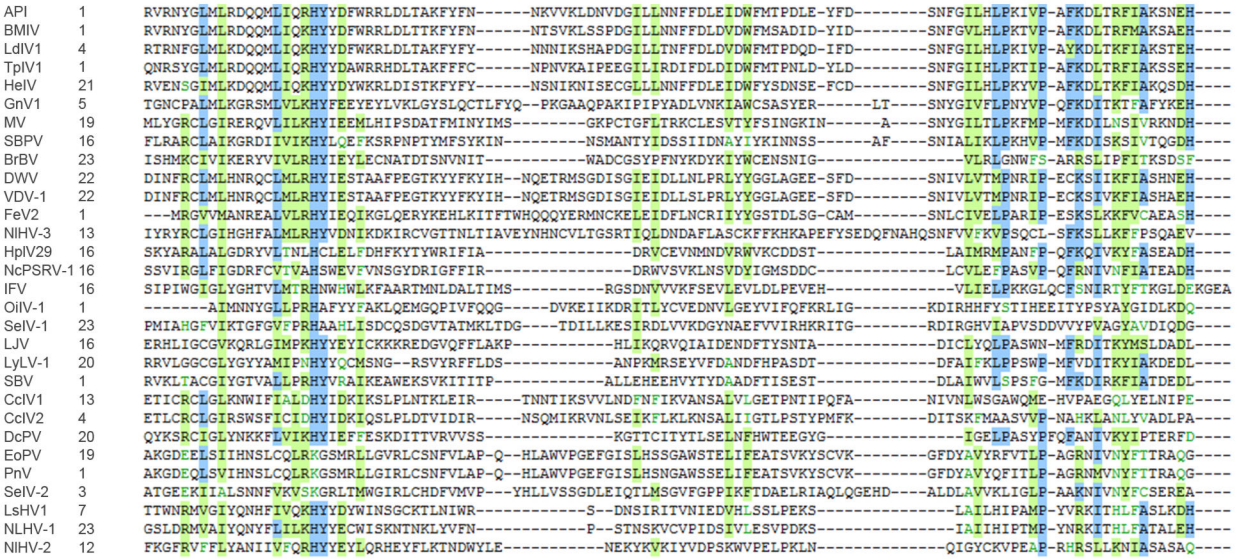
API 1 RVRNYGLMLRDQQMLIQRHYYDFWRRLDLTAKFYFN------NKVVKLDNVDGILLNNFFDLEIDWFMTPDLE-YFD--------SNFGILHLPKIVP-AFKDLTRFIAKSNEH----
BMIV 1 RVRNYGLMLRDQQMLIQKHYYDFWRRLDLTTKFYFN------NTSVKLSSPDGILLNNFFDLDVDWFMSADID-YID--------SNFGVLHLPKTVP-AFKDLTRFMAKSAEH----
LdIV1 4 RTRNFGLMLKDQQMLIQKHYYDFWKRLDLTAKFYFY------NNNIKSHAPDGILLTNFFDLDVDWFMTPDQD-IFD--------SNFGILHLPKIVP-AYKDLTKFIAKSTEH----
TpIV1 1 QNRSYGLMLRDQQMLIQRHYYDAWRRHDLTAKFFFC------NPNVKAIPEEGILIRDIFDLDIDWFMTPNLD-YLD--------SNFGIIHLPKTIP-AFKDLTRFIAKSSEH----
HeIV 21 RVENSGIMLKDQQMLIQKHYYDWKRLDISTKFYFY------NSNIKNISECGLLLNNFFDLEIDWFYSDNSE-FCD--------SNFGILHLPKYVP-AFKDLTKFIAKQSDH----
GnV1 5 TGNCPALMLKGRSMLVLKHYFEEYEYLVKLGYSLQCTLFYQ--PKGAAQPAKIPIPYADLVNKIAWCSASYER------LT----SNYGIVFLPNYVP-QFKDITKTFAFYKEH----
MV 19 MLYGRCLGIRERQVLILKHYIEEMLHIPSDATFMINYIMS--------GKPCTGFLTRKCLESVTYFSINGKIN------A----SNYGILTLPKFMP-MFKDILNSIVRKNDH----
SBPV 16 FLRARCLAIKGRDIIVIKHYLQEFKSRPNPTYMFSYKIN---------NSMANTYIDSSIIDNAYIYKINNSS------AF----SNIALIKLPKHVP-MFKDISKSIVTQGDH----
BrBV 23 ISHMKCIVIKERYVIVLRHYIEYLECNATDTSNVNIT----------WADCGSYPFNYKDYKIYWCENSNIG----------------VLRLGNWFS-ARRSLIPFITKSDSF----
DWV 22 DINFRCLMLHNRQCLMLRHYIESTAAFPEGTKYYFKYIH--NQETRMSGDISGIEIDLLNLPRLYYGGLAGEE-SFD--------SNIVLVTMPNRIP-ECKSIIKFIASHNEH----
VDV-1 22 DINFRCLMLHNRQCLMLRHYIESTAAFPEGTKYYFKYIH--NQETRMSGDISGIEIDLLSLPRLYYGGLAGEE-SFD--------SNIVLVTMPNRIP-ECKSIVKFIASHAEH----
FeV2 1 ---MRGVVMANREALVLRHYIEQIKGLQERYKEHLKITFTWHQQQYERMNCKELEIDFLNCRIIYYGSTDLSG-CAM--------SNLCIVELPARIP-ESKSLKKFVCAEASH----
NlHV-3 13 IYRYRCLGIHGHFALMLRHYVDNIKDKIRCVGTTNLTIAVEYNHNCVLTGSRTIQLDNDAFLASCKFFKHKAPEFYSEDQFNAHQSNFVVFKVPSQCL-SFKSLLKFFPSQAEV----
HpIV29 16 SKYARALALGDRYVLTNLHCLELFDHFKYTYWRIFIA----------------DRVCEVNMNDVRWVKCDDST-------------LAIMRMPANFP-QFKQIVKYFASEADH----
NcPSRV-1 16 SSVIRGLFIGDRFCVIVAHSWEVFVNSGYDRIGFFIR----------------DRWVSVKLNSVDYIGMSDDC-------------LCVLEFPASVP-QFRNIVNFIATEADH----
IFV 16 SIPIWGIGLYGHTVLMTRHNWHWLKFAARTMNLDALTIMS----------RGSDNVVVKFSEVLEVLDLPEVEH--------------VLIELPKKGLQCFSNIRTYFTKGLDEKGEA
OiIV-1 1 -------AIMNNYGLLPRHAFYYFAKLQEMGQPIVFQQG-----DVKEIIKDRITLYCVEDNVLGEYVIFQFKRLIG--------KDIRHHFYSTIHEEITYPSYAYGIDLKDQ----
SeIV-1 23 PMIAHGFVIKTGFGVFPRHAAHLISDCQSDGVTATMKLTDG----TDILLKESIRDLVVKDGYNAEFVVIRHKRITG--------RDIRGHVIAPVSDDVVYPVAGYAVDIQDG----
LJV 16 ERHLIGCGVKQRLGIMPKHYYEYICKKKREDGVQFFLAKP----------HLIKQRVQIAIDENDFTYSNTA-------------DICLYQLPASWN-MFRDITKYMSLDADL----
LyLV-1 20 RRVLGGCGLYGYYAMIPNHYYQCMSNG--RSVYRFFLDS----------ANPKMRSEYVFDANDFHPASDT-------------DFAIFKLPPSWP-MFVDITKYIAKDEDL----
SBV 1 RVKLTACGIYGTVALLPRHYVRAIKEAWEKSVKITITP----------ALLEHEEHVYTYDAADFTISEST-------------DLAIWVLSPSFG-MFKDIRKFIATDEDL----
CcIV1 13 ETICRCLGLKNWIFIALDHYIDKIKSLPLNTKLEIR-----TNNTIKSVVLNDFNFIKVANSALVLGETPNTIPQFA--------NIVNLWSGAWQME-HVPAEGQLYELNIPE----
CcIV2 4 ETLCRCLGIRSWSFICIDHYIDKIQSLPLDTVIDIR-----NSQMIKRVNLSEIKFLKLKNSALIIGTLPSTYPMFK--------DITSKFMAASVVP-NAHKLANLYVADLPA----
DcPV 20 QYKSRCIGLYNKKFLVIKHYIEFFESKDITTVRVVSS----------KGTTCITYTLSELNFHWTEEGYG---------------IGELPASYPFQFANIVKYIPTERFD----
EoPV 19 AKGDEELSIIHNSLCQLRKGSMRLLGVRLCSNFVLAP-Q--HLAWVPGEFGISLHSSGAWSTELIFEATSVKYSCVK--------GFDYAVYRFVTLP-AGRNIVNYFTTRAQG----
PnV 1 AKGDEQLSVIHNSLCQLRKGSMRLLGIRLCSNFVLAP-Q--HLAWVPGEFGISLHSNGAWSSELIFEATSVKYSCVK--------GFDYAVYQFITLP-AGRNMVNYFTTRAQG----
SeIV-2 3 ATGEEKIIALSNNFVKVSKGRLTMWGIRLCHDFVMVP---YHLLVSSGDLEIQTLMSGVFGPPIKFTDAELRIAQLQGEHD----ALDLAVVKLIGLP-AAKNIVNYFCSEREA----
LsHV1 7 TTWNRMVGIYQNHFIVQKHYYDYWINSGCKTLNIWR----------S--DNSIRITVNIEDVHLSSLPEKS--------------LAILHIPAMP-YVRKITHLFASLKDH----
NLHV-1 23 GSLDRMVAIYQNYFLILKHYYECWISKNTNKLYVFN----------P--STNSKVCVPIDSIVLESVPDKS--------------IAIIHIPTMP-YNRKITHLFATALEH----
NlHV-2 12 FKGFRVFFLYANIIVFQRHYYEYLQRHEYFLKTNDWYLE----------NEKYKVKIYVDPSKWVPELPKLN--------------QIGYCKVPEAP-RHRSLLKNIASASAQ----

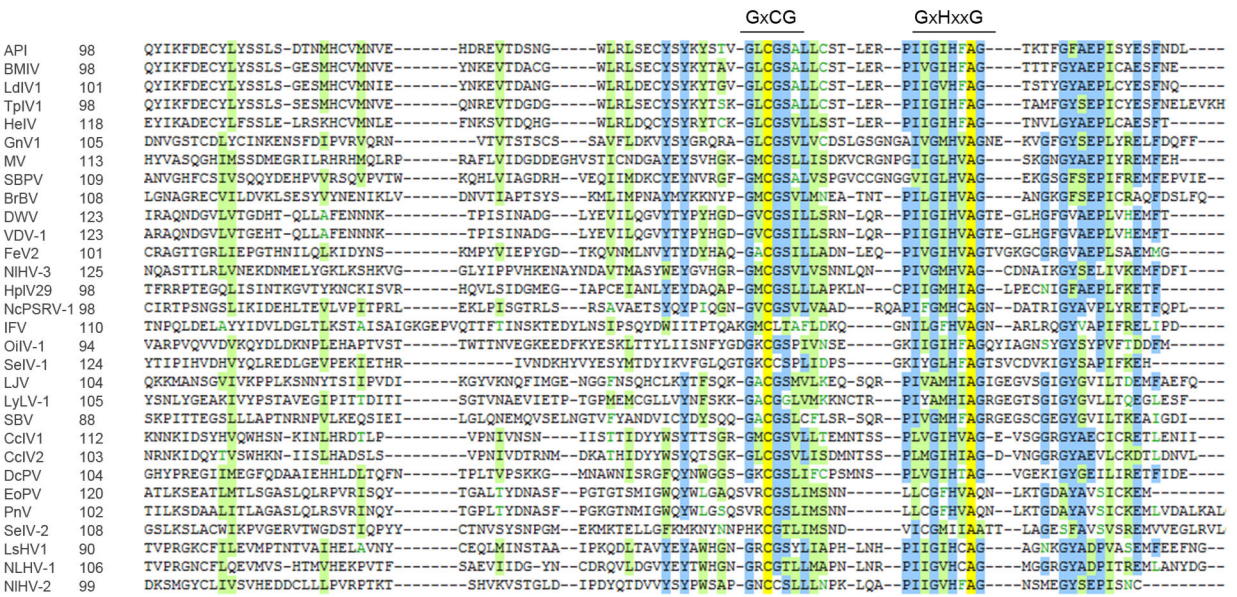
GxCG
GxHxxG
API 98 QYIKFDECYLYSSLS-DTNMHCVMNVE-------HDREVTDSNG-----WLRLSECYSYKYSTV-GLCGSALLCST-LER--PIIGIHFAG----TKTFGFAEPISYESFNDL----
BMIV 98 QYIKFDECYLYSSLS-GESMHCVMNVE-------YNKEVTDACG-----WLRLSECYSYKYTAV-GLCGSALLCST-LER--PIVGIHFAG----TTTFGYAEPICAESFNE-----
LdIV1 101 QYIKFDECYLYSSLS-GESMHCVMNIE-------YNKEVTDANG-----WLRLDECYSYKYTGV-GLCGSALLCST-LER--PIIGVHFAG----TSTYGYAEPLCYESFNQ-----
TpIV1 98 QYIKFDECYLYSSLS-SESMHCVMNVE-------QNREVTDGDG-----WLRLSECYSYKYTSK-GLCGSALLCST-LER--PIIGIHFAG----TAMFGYSEPICYESFNELEVKH
HeIV 118 EYIKADECYLFSSLE-LRSKHCVMNLE-------FNKSVTDQHG-----WLRLDQCYSYRYTCK-GLCGSVLLSST-LER--PIIGIHFAG----TNVLGYAEPLCAESFT------
GnV1 105 DNVGSTCDLYCINKENSFDIPVRVQRN---------VTVTSTSCS---SAVFLDKVYSYGRQRA-GLCGSVLVCDSLGSGNGAIVGMHVAGNE--KVGFGYSEPLYRELFDQFF---
MV 113 HYVASQGHIMSSDMEGRILRHRHMQLRP------RAFLVIDGDDEGHVSTICNDGAYEYSVHGK-GMCGSLLISDKVCRGNPGIIGLHVAG----SKGNGYAEPIYREMFEH-----
SBPV 109 ANVGHFCSIVSQQYDEHPVVRSQVPVTW------KQHLVIAGDRH--VEQIIMDKCYEYNVRGF-GMCGSALVSPGVCCGNGGVIGLHVAG----EKGSGFSEPIFREMFEPVIE--
BrBV 108 LGNAGRECVILDVKLSESVYNENIKLV------DNVTIAPTSYS---KMLIMPNAYMYKKNYP-GMCGSVLMNEA-TNT--PILGIHVAG----ANGKGFSEPICRAQFDSLFQ--
DWV 123 IRAQNDGVLVTGDHT-QLLAFENNNK---------TPISINADG---LYEVILQGVYTYPYHGD-GVCGSILLSRN-LQR--PIIGIHVAGTE-GLHGFGVAEPLVHEMFT------
VDV-1 123 ARAQNDGVLVTGEHT-QLLAFENNNK---------TPISINADG---LYEVILQGVYTYPYHGD-GVCGSILLSRN-LQR--PIIGIHVAGTE-GLHGFGVAEPLVHEMFT------
FeV2 101 CRAGTTGRLIEPGTHNILQLKIDYNS--------KMPYVIEPYGD--TKQVNMLNVYTYDYHAQ-GACGSILLADN-LEQ--PIVGIHVAGTVGKGCGRGVAEPLSAEMMG------
NlHV-3 125 NQASTTLRLVNEKDNMELYGKLKSHKVG------GLYIPPVHKENAYNDAVTMASYWEYGVHGR-GMCGSVLVSNNLQN---PIVGMHVAG--CDNAIKGYSELIVKEMFDFI----
HpIV29 98 TFRRPTEGQLISINTKGVTYKNCKISVR------HQVLSIDGMEG--IAPCEIANLYEYDAQAP-GMCGSLLLAPKLN---CPIIGMHIAG--LPECNIGFAEPLFKETF-------
NcPSRV-1 98 CIRTPSNGSLIKIDEHLTEVLVPITPRL------EKLPISGTRLS---RSAVAETSYQYPIQGN-GVCGSVLVAAD---RQAPIFGMHCAGN--DATRIGYAVPLYRETFQPL----
IFV 110 TNPQLDELAYYIDVLDGLTLKSTAISAIGKGEPVQTTFTINSKTEDYLNSIPSQYDWIITPTQAKGMCLTAFLDKQ-----GNILGFHVAGN--ARLRQGYVAPIFRELIPD-----
OiIV-1 94 VARPVQVVDVKQYDLDKNPLEHAPTVST------TWTTNVEGKEEDFKYESKLTTYLIISNFYGDGKCGSPIVNSE-----GKIIGIHFAGQYIAGNSYGYSYPVFTDDFM------
SeIV-1 124 YTIPIHVDHVYQLREDLGEVPEKIETHR----------IVNDKHYVYESYMTDYIKVFGLQGTGKCCSPLIDPS-----GKIYGLHFAGTSVCDVKIGYSAPIFKEH--------
LJV 104 QKKMANSGVIVKPPLKSNNYTSIIPVDI------KGYVKNQFIMGE-NGGFNSQHCLKYTFSQK-GACGSMVLKEQ-SQR--PIVAMHIAGIGEGVSGIGYGVILTDEMFAEFQ---
LyLV-1 105 YSNLYGEAKIVYPSTAVEGIPITTDITI------SGTVNAEVIETP-TGPMEMCGLLVYNFSKK-GACGGLVMKKNCTR---PIYAMHIAGRGEGTSGIGYGVLLTQEGLESF----
SBV 88 SKPITTEGSLLLAPTNRNPVLKEQSIEI------LGLQNEMQVSELNGTVFYANDVICYDYSQQ-GACGSLCFLSR-SQR--PIVGMHFAGRGEGSCGEGYGVILTKEAIGDI----
CcIV1 112 KNNKIDSYHVQWHSN-KINLHRDTLP---------VPNIVNSN----IISTTIDYYWSYTTSGR-GMCGSVLLTEMNTSS--PLVGIHVAG-E-VSGGRGYAECICRETLENII---
CcIV2 103 NRNKIDQYTVSWHKN-IISLHADSLS---------VPNIVDTRNM--DKATHIDYYWSYQTSGK-GLCGSVLISDMNTSS--PLMGIHIAG-D-VNGGRGYAEVLCKDTLDNVL---
DcPV 104 GHYPREGIIMEGFQDAAIEHHLDLTQFN------TPLTVPSKKG---MNAWNISRGFQYNWGGS-GKCGSLIFCPSMNS---PLVGIHTAG---VGEKIGYGEILIRETFIDE----
EoPV 120 ATLKSEATLMTLSGASLQLRPVRISQY-------TGALTYDNASF--PGTGTSMIGWQYWLGAQSVRCGSLIMSNN------LLCGFHVAQN--LKTGDAYAVSICKEM--------
PnV 102 TILKSDAALITLAGASLQLRSVRINQY-------TGPLTYDNASF--PGKGTNMIGWQYWLGSQSVRCGSLIMSNN------LLCGFHVAQN--LKTGDAYAVSICKEMLVDALKAL
SeIV-2 108 GSLKSLACWIKPVGERVTWGDSTIQPYY------CTNVSYSNPGM--EKMKTELLGFKMKNYNNPHKCGTLIMSND------VICGMIIAATT--LAGESFAVSVSREMVVEGLRVL
LsHV1 90 TVPRGKCFILEVMPTNTVAIHELAVNY-------CEQLMINSTAA--IPKQDLTAVYEYAWHGN-GRCGSYLIAPH-LNH--PIIGIHCAG----AGNKGYADPVASEMFEEFNG--
NLHV-1 106 TVPRGNCFLQEVMVS-HTMVHEKPVTF-------SAEVIIDG-YN--CDRQVLDGVYEYTWHGN-GRCGTLLMAPN-LNR--PIIGVHCAG----MGGRGYADPITREMLANYDG--
NlHV-2 99 DKSMGYCLIVSVHEDDCLLLPVRPTKT--------SHVKVSTGLD--IPDYQTDVVYSYPWSAP-GNCCSLLLNPK-LQA--PIIGVHFAG----NSMEGYSEPISNC---------

F RdRp

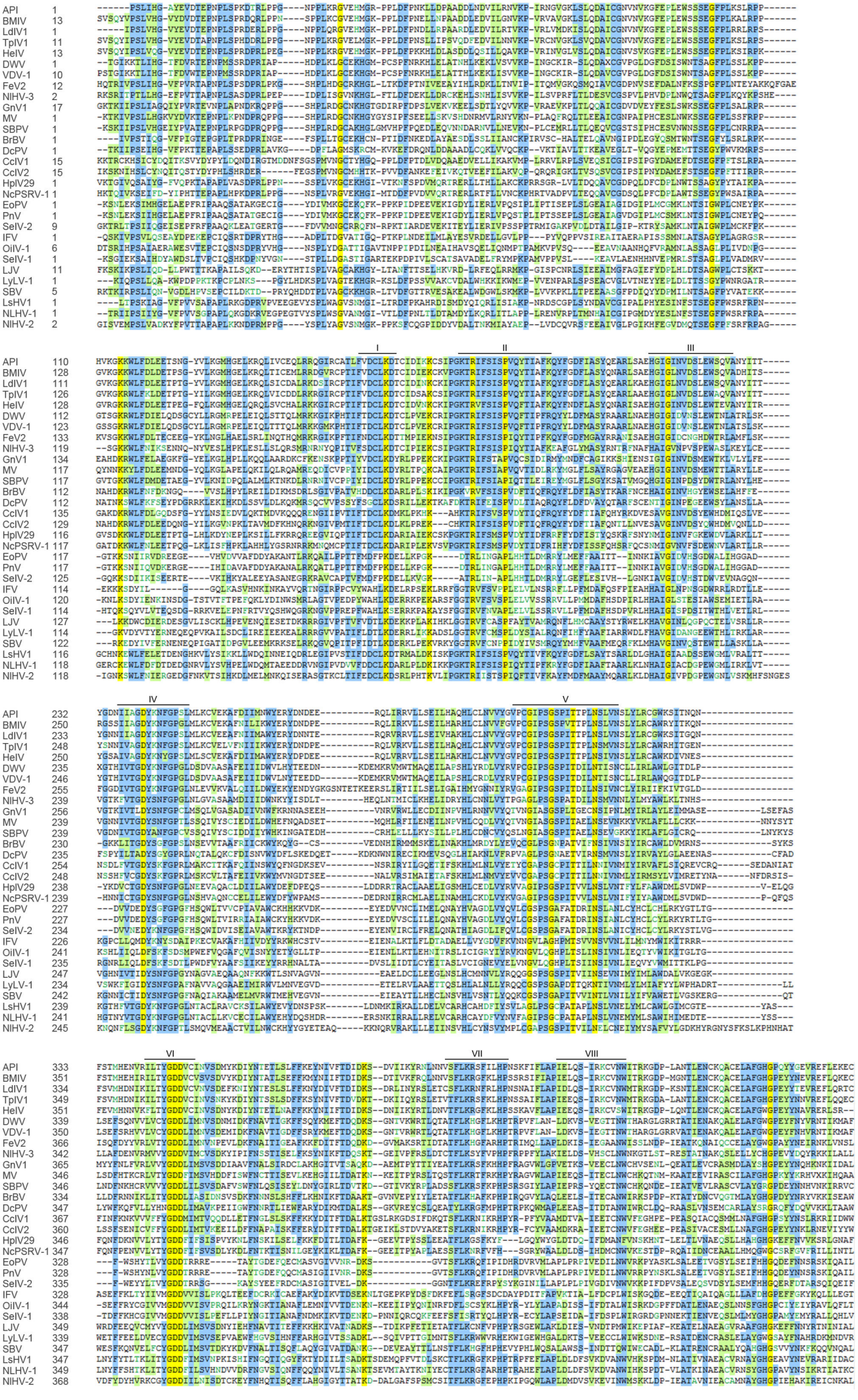
API
BMIV
LdIV1
TpIV1
HeIV
DWV
VDV-1
FeV2
NIHV-3
GnV1
MV
SBPV
BrBV
DcPV
CcIV1
CcIV2
HpIV29
NcPSRV-1
EoPV
PnV
SeIV-2
IFV
OiIV-1
SeIV-1
LJV
LyLV-1
SBV
LsHV1
NLHV-1
NIHV-2
I
II
III
IV
V
VI
VII
VIII
