# Supplementary material for: A Novel Iflavirus Was Discovered in Green Rice Leafhopper Nephotettix cincticeps and Its Proliferation Was Inhibited by Infection of Rice Dwarf Virus
Source: Front Microbiol. 2021 Jan 8;11:621141. doi: 10.3389/fmicb.2020.621141 (PMC7820178; doi:10.3389/fmicb.2020.621141)

A *N. apicalis*

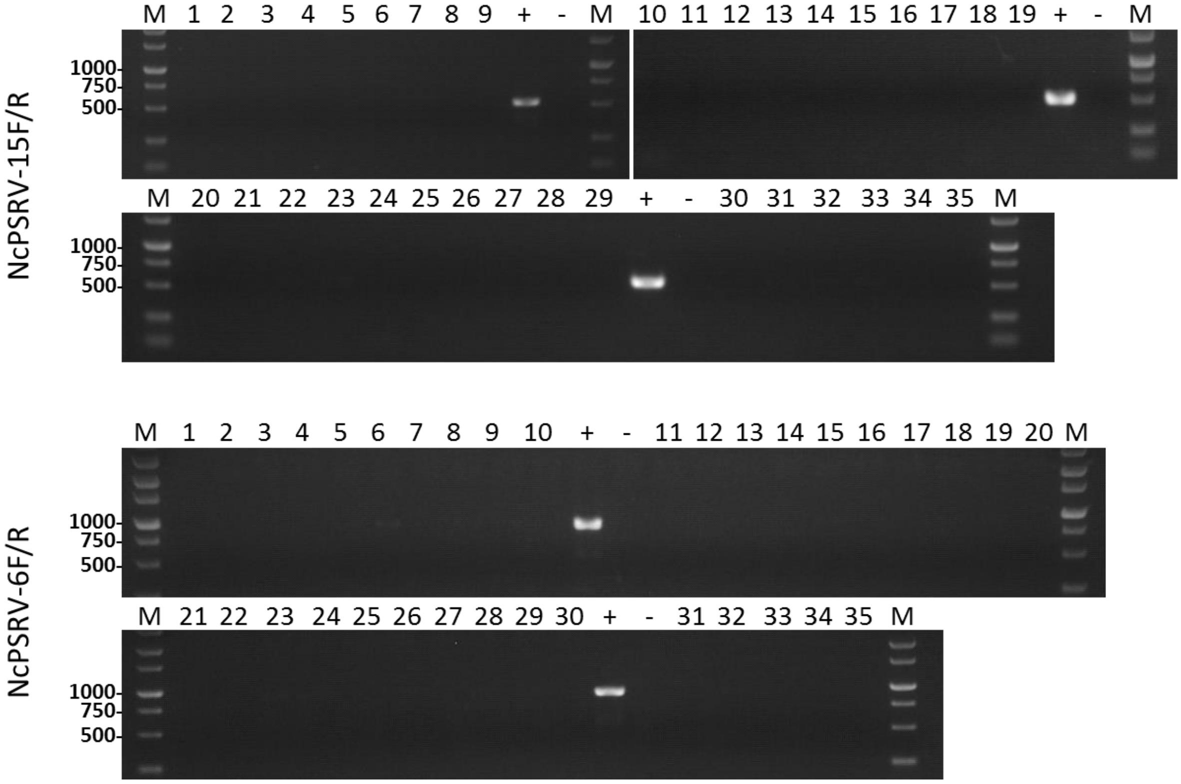

B *N.apicalis* 12 dpi

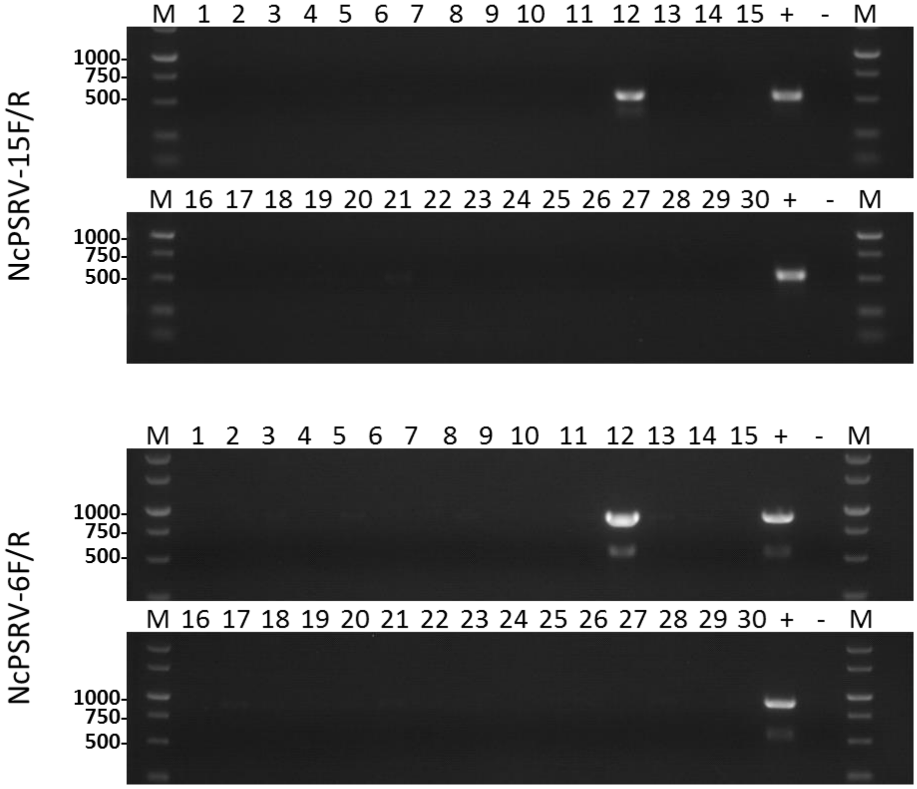

C *R. dorsalis*

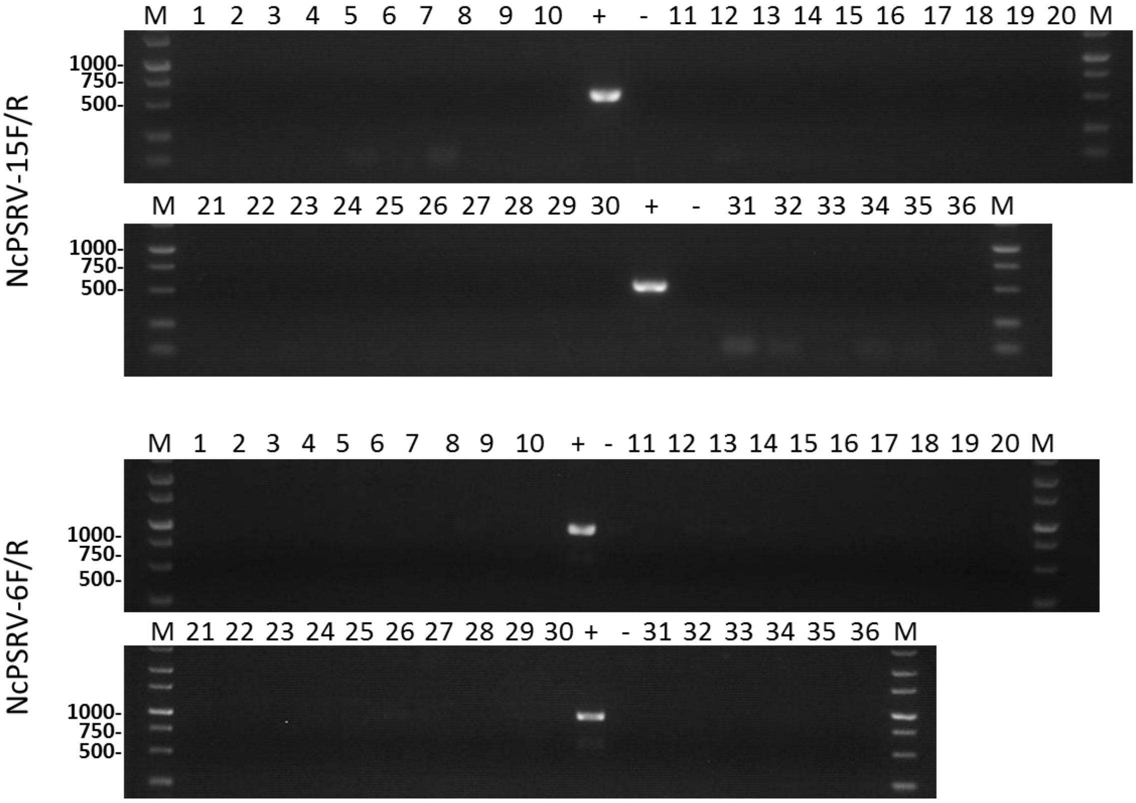

D *R.dorsalis* 12 dpi

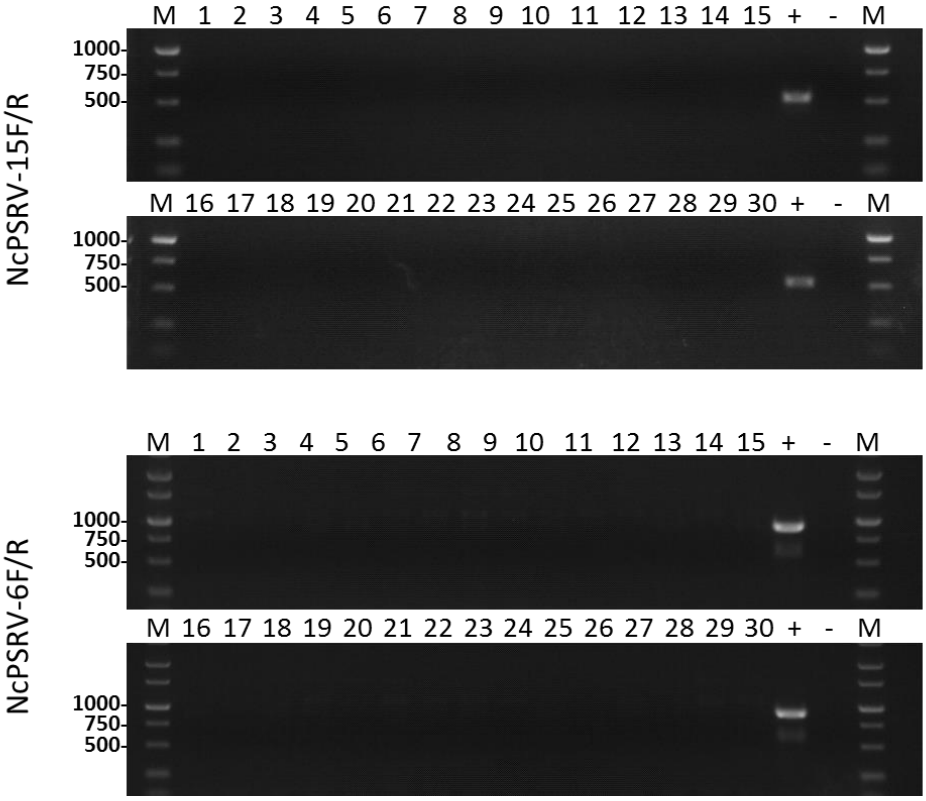

E *O. sativa* strain TN1

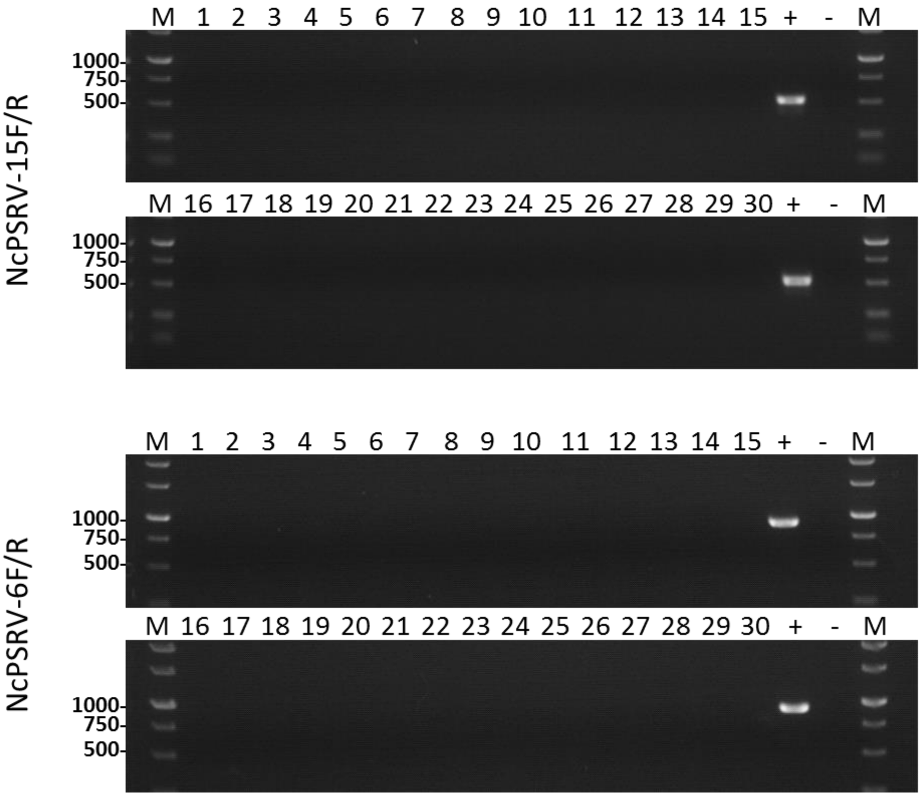

F *O. sativa* strain TN1 12 dpi

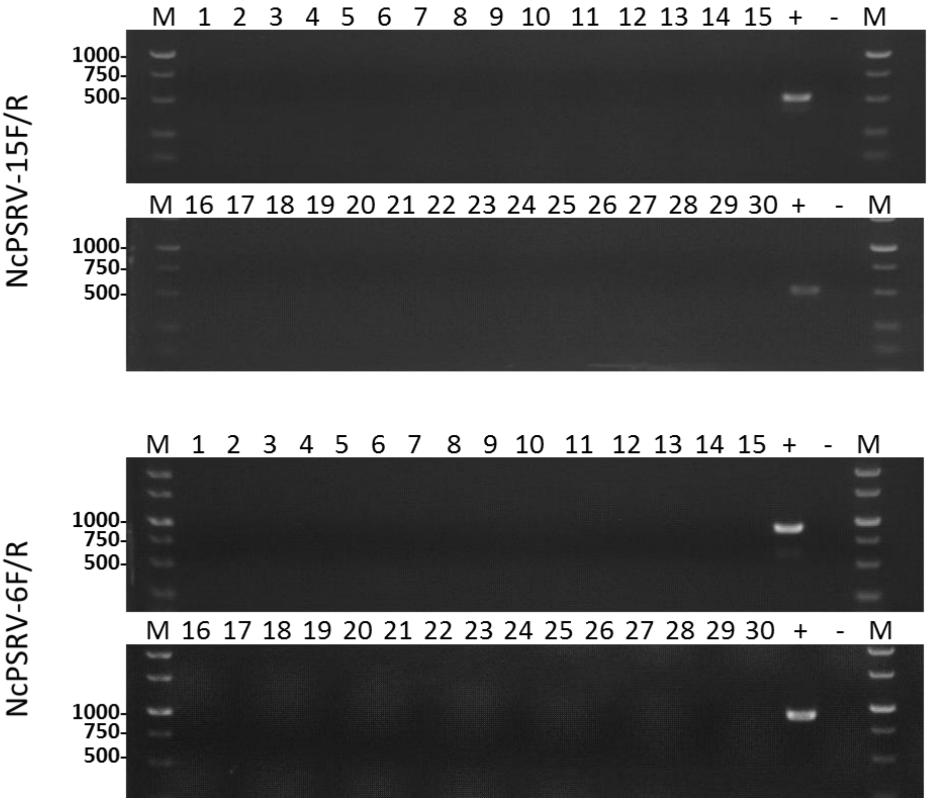

Supplement: Supplementary file 1 [file Data_Sheet_1.zip › Supplementary Material Presentation/Supplementary Figure S6.pdf]
