# Supplementary material for: A Novel Iflavirus Was Discovered in Green Rice Leafhopper Nephotettix cincticeps and Its Proliferation Was Inhibited by Infection of Rice Dwarf Virus
Source: Front Microbiol. 2021 Jan 8;11:621141. doi: 10.3389/fmicb.2020.621141 (PMC7820178; doi:10.3389/fmicb.2020.621141)
Supplement: Supplementary file 1 [file Data_Sheet_1.zip › Supplementary Material Presentation/Supplementary Material.docx]

Supplementary Material

**Supplementary Figure S1.** The eggs of *N. cincticeps*.

**Supplementary Figure S2.** PCR amplication of NcPSRV-1 in *N. cincticeps*. (-) means negative control. The amplified bands in lanes 1-15 are corresponding to the fifteen pairs of primers listed in Supplementary Table S1. The eggs of *N. cincticeps*.

**Supplementary Figure S3.** protein domain alignments of NcPSRV-1 and selected iflaviruses. **(A)** vp2; **(B)** vp3; **(C)** vp1; **(D)** helicase; **(e)** protease; **(f)** RdRp. In **(A) (C)**, identical residues are shaded in orange, conservative residues in purple, similar residues in light purple and weakly similar residues in grey. In **(B)**, identical residues are shaded in orange, conservative residues in blue, similar residues in light purple and weakly similar residues in grey. In **(D) (e) (f)**, identical residues are shaded in yellow, conservative residues in blue, similar residues in green and weakly similar residues are in green text. Numbers in front of the sequences represent the amino acid positions from the N-terminus of the viral polyprotein. Conserved motifs are marked with black bars. The viral abbreviations and accession numbers are listed in Supplementary Table S2. The iflavirus conserved motifs in capsid proteins proposed in the study are listed as follows. The VP2 contained motif I [N(V/T)(V/L/I) (V/L/I)T], motif II [(S/T) (S/T) (D/E)x_7_Lx_2_RWx_7-10_W], motif III [(I/L)x_4_LP], motif IV [Px_3_PFx_8_D(I/M)x_5_(I/V)N(A/S)Nx_2_QxGxLx_5_Y], motif V [Hx_2_(I/L)x_5_Nx_3_Lx(I/V)P(Y/F)], motif VI [(L/V)x_3_VLxPL] and motif VII [(V/L/I)x_3_FxNx_2_FxG]. The VP3 contained motif I [Px_4_Sx_3_Gx_6-7_LRL], motif II [(I/V/L)x_4_(G/M)Lx_5_W], motif III [Gx_2_Lx_6_P], motif IV [(P/T)Px_4_Sx_5_(W/Y)xGx(L/I)x_10_H(T/S/N)Gx(L/V/I)x(L/V/I)x(Y/F)xP], motif V [(V/I)PY] and motif VI [V(L/V)NxLx_9_(I/V)x(I/V)x_4_(R/A)(G/A)(G/A)x_2_(F/M)x_4_P]. The VP1 contained motif I [G x_3_FGExFxDLKDx_2_RRYQxY], motif II [Px_0-7_PxGL], motif III [R(E/D)GxIP(L/V/I)(I/L/V)xSx (Y/F)R(F/Y)(Y/F)RGxLRxR(I/L/V)(V/I)], motif IV [W(Y/F)(V/I)QHxP], motif V [Nx_2_Yx_4_Qx_3_ (V/L)NN(V/I)(V/I/L)(E/S)(I/L/V)EVPFY], motif VI [LGx(L/I/V)x(I/V/L)G] and motif VII [(I/V)xY(S/A)x(A/G)DDx_5_(F/Y/W)xGxPP(M/L/V)].

**Supplementary Figure S4.** Horizontal transmission detection of NcPSRV-1 in *N. cincticeps*. **(A)** PCR detection of NcPSRV-1 in *N. cincticeps* individuals 12 days after oral inoculation with crude virus filtrate. **(B)** PCR detection of NcPSRV-1 in *N. cincticeps* individuals without oral inoculation. (+) means positive control. (-) means negative control. Lanes 1-38 correspond to 38 individuals tested.

**Supplementary Figure S5.** Vertical transmission model detection of NcPSRV-1 in *N. cincticeps*. PCR detection of NcPSRV-1 in *N. cincticeps* offsprings of the four mated pairs: **(A)** ♀+/♂- and ♀-/♂+; **(B)** ♀+/♂+; **(C)** ♀-/♂-. (+) means positive control. (-) means negative control.

**Supplementary Figure S6.** Host range detection of NcPSRV-1. **(A)** PCR detection of NcPSRV-1 in *N. apicalis* individuals. **(B)** PCR detection of NcPSRV-1 in *N. apicalis* individuals 12 days after oral inoculation. **(C)** PCR detection of NcPSRV-1 in *R. dorsals* individuals. **(D)** PCR detection of NcPSRV-1 in *R. dorsalis* individuals 12 days after oral inoculation. **(E)** PCR detection of NcPSRV-1 in *O. sativa* strain TN1 individuals. **(F)** PCR detection of NcPSRV-1 in *O. sativa* strain TN1 individuals 12 days after the feeding of NcPSRV-1 positive *N. cincticeps*. (+) means positive control. (-) means negative control.
