# Supplementary material for: A Novel Iflavirus Was Discovered in Green Rice Leafhopper Nephotettix cincticeps and Its Proliferation Was Inhibited by Infection of Rice Dwarf Virus
Source: Front Microbiol. 2021 Jan 8;11:621141. doi: 10.3389/fmicb.2020.621141 (PMC7820178; doi:10.3389/fmicb.2020.621141)
Supplement: Supplementary file 1 [file Data_Sheet_1.zip › Supplementary Material Presentation/Supplementary Table S1.docx]

**Supplementary Table S1. Primers Used in This Study**

| **Primers** | **Genome positions** | **Oligonucleotide (5'-3')** | **Orientation** | **Application** |
| --- | --- | --- | --- | --- |
| NcPSRV-1F | 370-389 | GAACCTGTCTGTTCGGGCAG | → | Verification of viral genome sequence |
| NcPSRV-1R | 1245-1265 | GTCAATGCGCATCTCTACGGT | ← |  |
| NcPSRV-2F | 1066-1089 | TAATTAAGGAGAAGAGGCGAGAGG | → |  |
| NcPSRV-2R | 1931-1955 | GGAGGCAACATTCATTCTATTAGCT | ← |  |
| NcPSRV-3F | 1787-1808 | TGGCACGCCTAATAGTGTTCCT | → |  |
| NcPSRV-3R | 2566-2587 | TGGTCCACCGTTGAAATTGAAT | ← |  |
| NcPSRV-4F | 2418-2439 | GCGCGTTACACTGATGCAGCCT | → |  |
| NcPSRV-4R | 3295-3319 | TCAGTCTTAACATCTGGTTGGCTCT | ← |  |
| NcPSRV-5F | 3185-3206 | TGAACACATAGCCCAAGTTTGG | → |  |
| NcPSRV-5R | 4017-4037 | TCCATCGACATAGGATGCGAT | ← |  |
| NcPSRV-6F | 3892-3912 | TGCTGTCAGGGTACCGTTTCT | → |  |
| NcPSRV-6R | 4767-4788 | GGTGTTGATCTAGGAGCGCTCT | ← |  |
| NcPSRV-7F | 4664-4689 | TCCTGTAGTTATGGCCATAGCAGTAG | → |  |
| NcPSRV-7R | 5524-5543 | TCCAGAACGCGTCACGAAAG | ← |  |
| NcPSRV-8F | 5423-5442 | TGCCCGTGTACCAATAACGG | → |  |
| NcPSRV-8R | 6288-6311 | GTTCGCTAGGAGACCCATTTTCAT | ← |  |
| NcPSRV-9F | 6143-6167 | TGAGATGAGTCCACCTGCTAAGATG | → |  |
| NcPSRV-9R | 6828-6851 | AGTGTCGACGAAGATGATGAATTA | ← |  |
| NcPSRV-10F | 6710-6733 | TAAGATCGATTGGGATAAGAAGCG | → |  |
| NcPSRV-10R | 7586-7609 | GGAGTAGTATGAATTACCGCGGCT | ← |  |
| NcPSRV-11F | 7470-7491 | GCTGCGACGATTAGGTTCATTA | → |  |
| NcPSRV-11R | 8326-8346 | TACGGCTAAGACGAGTTCCGC | ← |  |
| NcPSRV-12F | 8204-8225 | TATTGCCACTGAAGCCGATCAT | → |  |
| NcPSRV-12R | 9108-9129 | GATTCATCTTACGGCGGTTACG | ← |  |
| NcPSRV-13F | 8955-8975 | TTAGCGTGGACTTCGTCGGAG | → |  |
| NcPSRV-13R | 9855-9877 | GCGAGAGGAGCGTAAGGTGTAAT | ← |  |
| NcPSRV-14F | 9507-9525 | ATTCGGCGGTGTATGCTCG | → |  |
| NcPSRV-14R | 10385-10408 | TATCTTATGCAACTCAGGTCGCTC | ← |  |
| NcPSRV-15F | 9989-10008 | TCCAAGGCAAGCCATTATCG | → |  |
| NcPSRV-15R | 10476-10496 | GCTTAAGGCCTCCGGGGTTAT | ← |  |
| 5' RACE-GSP1 | 877-904 | CTAGGTTGGGGCCCGATGGGGAAGGCAT | ← | 5' RACE of 5' UTR |
| 5' RACE-GSP2 | 1314-1336 | CGTTTTGGTGGGAGACGGCCGCC | ← |  |
| 3' RACE-GSP1 | 9429-9456 | GTCGCGCAAAATTGCTGTGAGTTGATCC | → | 3' RACE of 3' UTR |
| 3' RACE-GSP2 | 9387-9414 | ACTGGCGACTACTCGAATTTCGGTCCTG | → |  |
| NcPSRV-1S | 1619-1637 | TACTCCTGCTGCTCCTTCC | → | Quantification of NcPSRV-1 |
| NcPSRV-1A | 1725-1744 | AATGACGCTCCTTGCTCTTC | ← |  |
| NcActin-1S |  | GTGTTGGATTCTGGTGATG | → | Reference gene |
| NcActin-1A |  | GGTAGTCTGTAAGGTCTCG | ← |  |
| RDV-S8-S |  | AACTATTCTCGCACTTCAG | → | Quantification of RDV |
| RDV-S8-A |  | ACCAGCAGATTCCTTATATG | ← |  |
