# Supplementary material for: A Novel Iflavirus Was Discovered in Green Rice Leafhopper Nephotettix cincticeps and Its Proliferation Was Inhibited by Infection of Rice Dwarf Virus
Source: Front Microbiol. 2021 Jan 8;11:621141. doi: 10.3389/fmicb.2020.621141 (PMC7820178; doi:10.3389/fmicb.2020.621141)
Supplement: Supplementary file 1 [file Data_Sheet_1.zip › Supplementary Material Presentation/Supplementary Table S2.docx]

**Supplementary Table S2. Viral Sequences Selected for Phylogenetic Analysis and Sequence Alignments in This Study**

| ***Picornavirales* family** | **Genus** | **Natural host** | **Virus** | **Virus abbreviation** | **RefSeq or GenBank accession number** |
| --- | --- | --- | --- | --- | --- |
| [*Picornaviridae*](https://viralzone.expasy.org/by_protein/33) | *Ampivirus* | Amphibians | Ampivirus A1 | AMV-A | NC_027214 |
|  | *Aphthovirus* | Mostly cloven-hooved animals | Foot-and-mouth disease virus | NDV | NC_039210 |
|  | *Aquamavirus* | Pinnipeds | Seal picornavirus | SPV | [NC_009891](https://www.ncbi.nlm.nih.gov/nuccore/NC_009891) |
|  | *Avihepatovirus* | Ducks and geese | Duck hepatitis A virus 1 | DHAV-1 | [NC_008250](https://www.ncbi.nlm.nih.gov/nuccore/NC_008250) |
|  | *Avisivirus* | Turkeys, chickens | Avisivirus A | AsV-A | KC465954 |
|  | *Cardiovirus* | Humans, vertebrates | Encephalomyocarditis virus | EMCV-1 | NC_001479 |
|  | *Cosavirus* | Humans, pigs | Cosavirus A1 | CoSV-A1 | NC_012800 |
|  | *Dicipivirus* | Dogs | Canine picodicistrovirus | CaPd | NC_021178 |
|  | *Enterovirus* | Humans, mammals | Coxsackievirus A2 | CVA-2 | NC_038306 |
|  |  |  | Poliovirus | PV-1 | [NC_002058](https://www.ncbi.nlm.nih.gov/nuccore/NC_002058) |
|  |  |  | Rhinovirus A1 | RV-A1 | NC_038311 |
|  | *Erbovirus* | Horses | Equine rhinitis B virus 1 | ERBV-1 | [NC_003983](https://www.ncbi.nlm.nih.gov/nuccore/NC_003983) |
|  | *Gallivirus* | Turkeys, chickens | Gallivirus A1 | GV-A1 | NC_018400 |
|  | *Harkavirus* | Kestrels | Falcovirus A1 | FaV | NC_026921 |
|  | *Hepatovirus* | Humans, vertebrates | Hepatitis A virus | HAV | NC_001489 |
|  | *Hunnivirus* | Cattle, sheep, rats | Hunnivirus A | HuV-A1 | NC_018668 |
|  | *Kobuvirus* | Humans, vertebrates | Aichi virus 1 | AiV-A1 | NC_001918 |
|  |  |  | Bovine kobuvirus | BKV | NC_004421 |
|  | *Kunsagivirus* | European rollers | Kunsagivirus A | KuV-A | NC_038317 |
|  | *Limnipivirus* | Fish | Bluegill picornavirus | BGPV | NC_018506 |
|  | *Megrivirus* | Birds | Turkey hepatitis virus | MeV-A2 | NC_021201 |
|  | *Mischivirus* | Bats | Miniopterus schreibersii picornavirus | MiV-A | NC_034381 |
|  | *Mosavirus* | Canyon mouse, european roller | Mosavirus A2 | MoV-A2 | KF958461 |
|  | *Oscivirus* | Birds | Oscivirus A1 | OsV-A1 | NC_014412 |
|  | *Parechovirus* | Humans, rodents | Human parechovirus 1 | HPeV-1 | NC_038319 |
|  | *Pasivirus* | Pigs | Pasivirus A1 | PaV-A1 | NC_018226 |
|  | *Passerivirus* | Thrushes | Passerivirus A1 | PasV-A | NC_014411 |
|  | *Potamipivirus* | Eels | Eel picornavirus 1 | EPV | NC_022332 |
|  | *Rabovirus* | Rats | Rabovirus A | RaBoV-A | NC_026314 |
|  | *Rosavirus* | Humans, rodents | Rosavirus A2 | RoV-A2 | NC_024070 |
|  | *Sakobuvirus* | Cats | Sakobuvirus A | SakV-A | NC_022802 |
|  | *Salivirus* | Humans, chimpanzees | Salivirus A1 | SaV-A1 | NC_012957 |
|  | *Sapelovirus* | Pigs | Porcine sapelovirus 1 | PSV | NC_003987 |
|  | *Senecavirus* | Pigs | Seneca Valley virus | SVV | NC_011349 |
|  | *Sicinivirus* | Birds | Sicinivirus A | SiV-A | NC_023861 |
|  | *Teschovirus* | Pigs | Teschovirus A | TV-A | NC_003985 |
|  | *Torchivirus* | Tortoises | Tortoise picornavirus | ToPV | NC_025890 |
|  | *Tremovirus* | Birds | Avian encephalomyelitis virus | AEV | NC_003990 |
| *Dicistroviridae* | *Aparavirus* | Arthropods | Acute bee paralysis virus | ABPV | NC_002548 |
|  | *Cripavirus* | Arthropods | Cricket paralysis virus | CrPV | NC_003924 |
|  | *Triatovirus* | Arthropods | Black queen cell virus | BQCV | NC_003784 |
| *Polycipiviridae* | *Chipolycivirus* | Arthropods | Chironomus riparius virus | ChriV1 | KA182589 |
|  | *Hupolycivirus* | Arthropods | Hubei picorna-like virus 81 | HplV81 | NC_033152 |
|  | *Sopolycivirus* | Arthropods | Solenopsis invicta virus 2 | SINV2 | NC_039236 |
| *Secoviridae* | *Cheravirus* | Plants | Cherry rasp leaf virus | CRLV | NC_006271 |
|  | *Sadwavirus* | Plants | Satsuma dwarf virus | SDV | NC_003785 |
|  | *Sequivirus* | Plants | Parsnip yellow fleck virus | PYFV | NC_003628 |
|  | *Torradovirus* | Plants | Tomato torrado virus | ToTV | NC_009013 |
|  | *Waikavirus* | Plants | Rice tungro spherical virus | RTSV | NC_001632 |
| *Iflaviridae* | *Iflavirus* | Arthropoda | Antheraea pernyi iflavirus | API | NC_023483 |
|  |  |  | Brevicoryne brassicae virus | BrBV | NC_009530 |
|  |  |  | Deformed wing virus | DWV | NC_004830 |
|  |  |  | Dinocampus coccinellae paralysis virus | DcPV | NC_025835 |
|  |  |  | Ectropis obliqua picorna-like virus | EoPV | NC_005092 |
|  |  |  | Infectious flacherie virus | IFV | NC_003781 |
|  |  |  | Lygus lineolaris virus 1 | LyLV-1 | NC_038301 |
|  |  |  | Lymantria dispar iflavirus 1 | LdIV1 | NC_024497 |
|  |  |  | Nilaparvata lugens honeydew virus 1 | NLHV-1 | NC_038302 |
|  |  |  | Perina nuda virus | PnV | NC_003113 |
|  |  |  | Sacbrood virus | SBV | NC_002066 |
|  |  |  | Slow bee paralysis virus | SBPV | NC_014137 |
|  |  |  | Spodoptera exigua iflavirus 1 | SeIV-1 | NC_016405 |
|  |  |  | Spodoptera exigua iflavirus 2 | SeIV-2 (SeV) | NC_023676 |
|  |  |  | Varroa destructor virus-1 | VDV-1 | NC_006494 |
|  |  |  | Bombyx mori iflavirus | BMIV | LC068762 |
|  |  |  | Ceratitis capitate iflavirus 1 | CcIV1 | GAMC01001920 |
|  |  |  | Ceratitis capitate iflavirus 2 | CcIV2 | GAMC01020602 |
|  |  |  | Formica exsecta virus 2 | FeV2 | KF500002 |
|  |  |  | Graminella nigrifrons virus 1 | GnV1 | KP866792 |
|  |  |  | Heliconius erato iflavirus | HeIV | NC_024016 |
|  |  |  | La Jolla virus | LJV | KP714074 |
|  |  |  | Laodelphax striatella honeydew virus 1 | LsHV1 | NC_023627 |
|  |  |  | Moku virus | MV | NC_031338 |
|  |  |  | Nilaparvata lugens honeydew virus-2 | NlHV-2 | AB826459 |
|  |  |  | Nilaparvata lugens honeydew virus-3 | NlHV-3 | AB826460 |
|  |  |  | Opsiphanes invirae iflavirus 1 | OiIV-1 | KR534892 |
|  |  |  | Thaumetopoea pityocampa iflavirus 1 | TpIV1 | KP217032 |
|  |  |  | Hubei picorna-like virus 29 | HplV29 | NC_032776 |
|  |  |  | Nephotettix cincticeps positive-stranded RNA virus-1 | NcPSRV-1 | MW197427 |

Virus names, the choice of exemplar isolates and virus abbreviations were based on ICTV, ViralZone and related references.
