# Supplementary figures and images for: A Novel Iflavirus Was Discovered in Green Rice Leafhopper Nephotettix cincticeps and Its Proliferation Was Inhibited by Infection of Rice Dwarf Virus
Source: Front Microbiol. 2021 Jan 8;11:621141. doi: 10.3389/fmicb.2020.621141 (PMC7820178; doi:10.3389/fmicb.2020.621141)

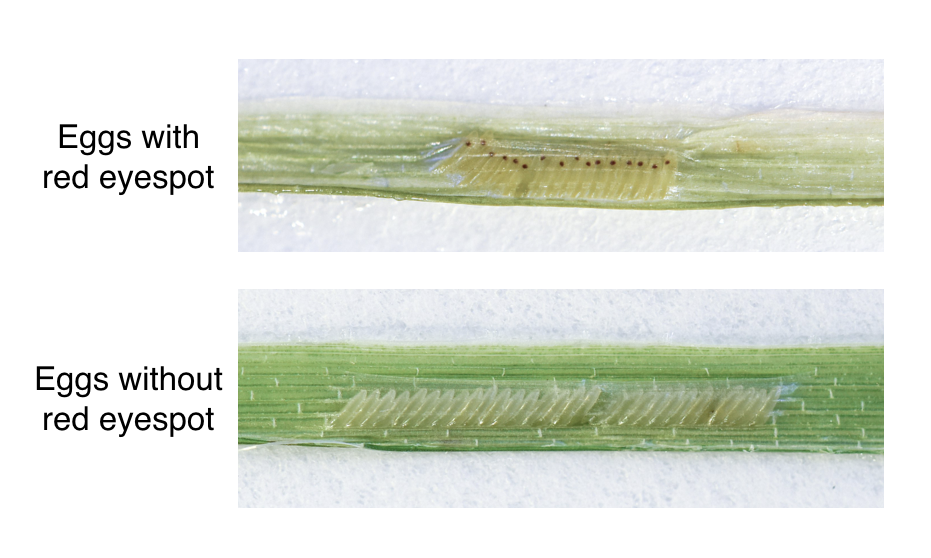

Supplement: Supplementary file 1 [file Data_Sheet_1.zip › Supplementary Material Presentation/Supplementary Figure S1.tiff]

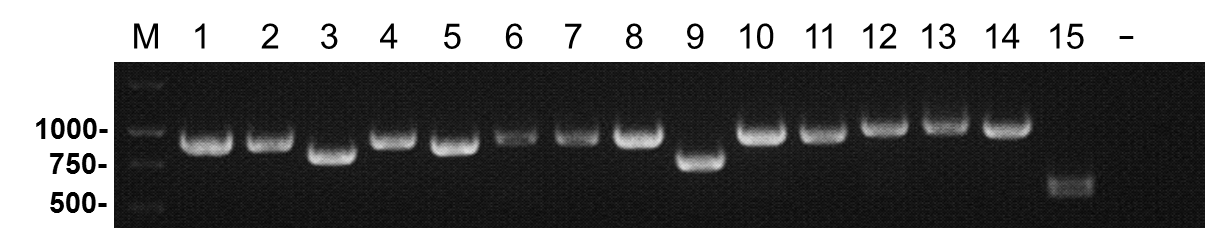

Supplement: Supplementary file 1 [file Data_Sheet_1.zip › Supplementary Material Presentation/Supplementary Figure S2.tif]

**A** *N. cincticeps* with oral inoculation

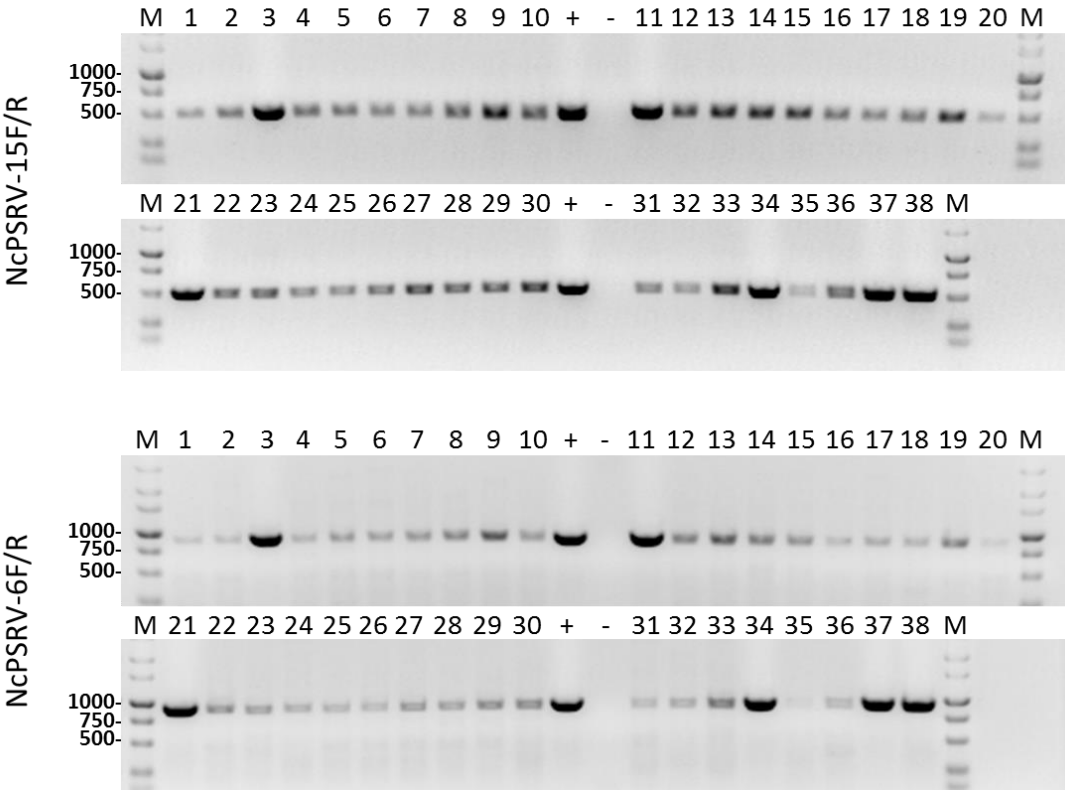

**B** *N. cincticeps* without oral inoculation

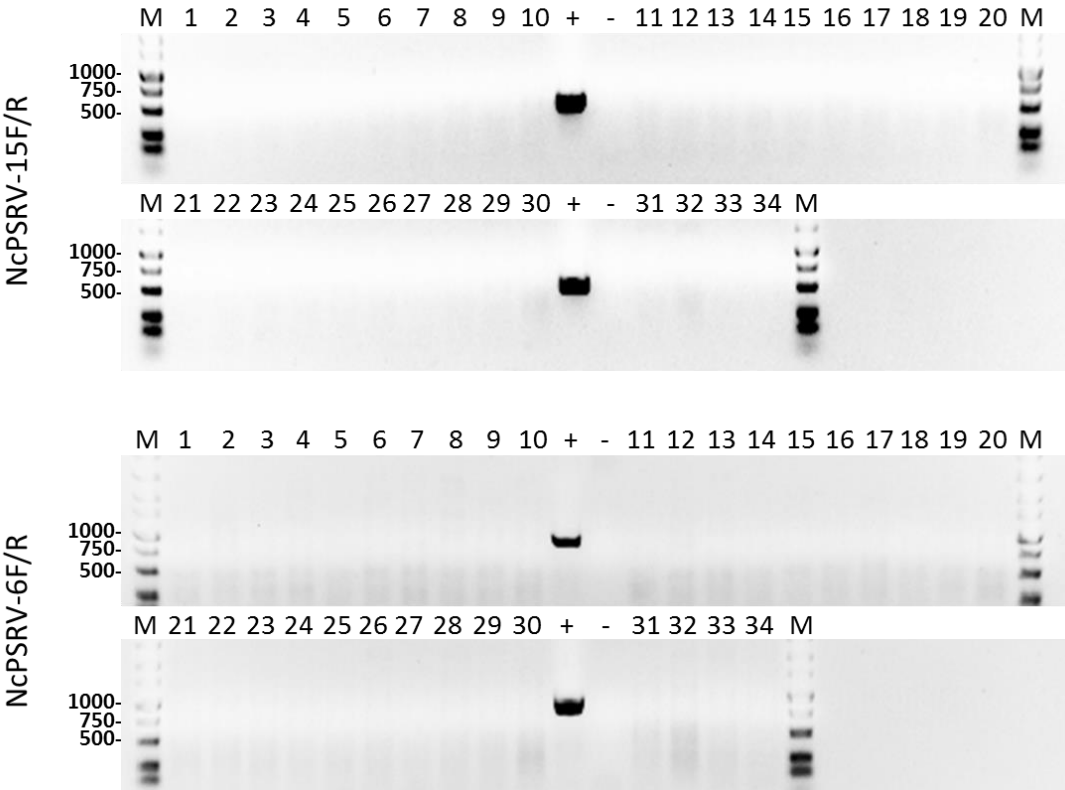

Supplement: Supplementary file 1 [file Data_Sheet_1.zip › Supplementary Material Presentation/Supplementary Figure S4.pdf]

**A** *N. cincticeps* ♀ +/ ♂ -, ♀ -/ ♂ +

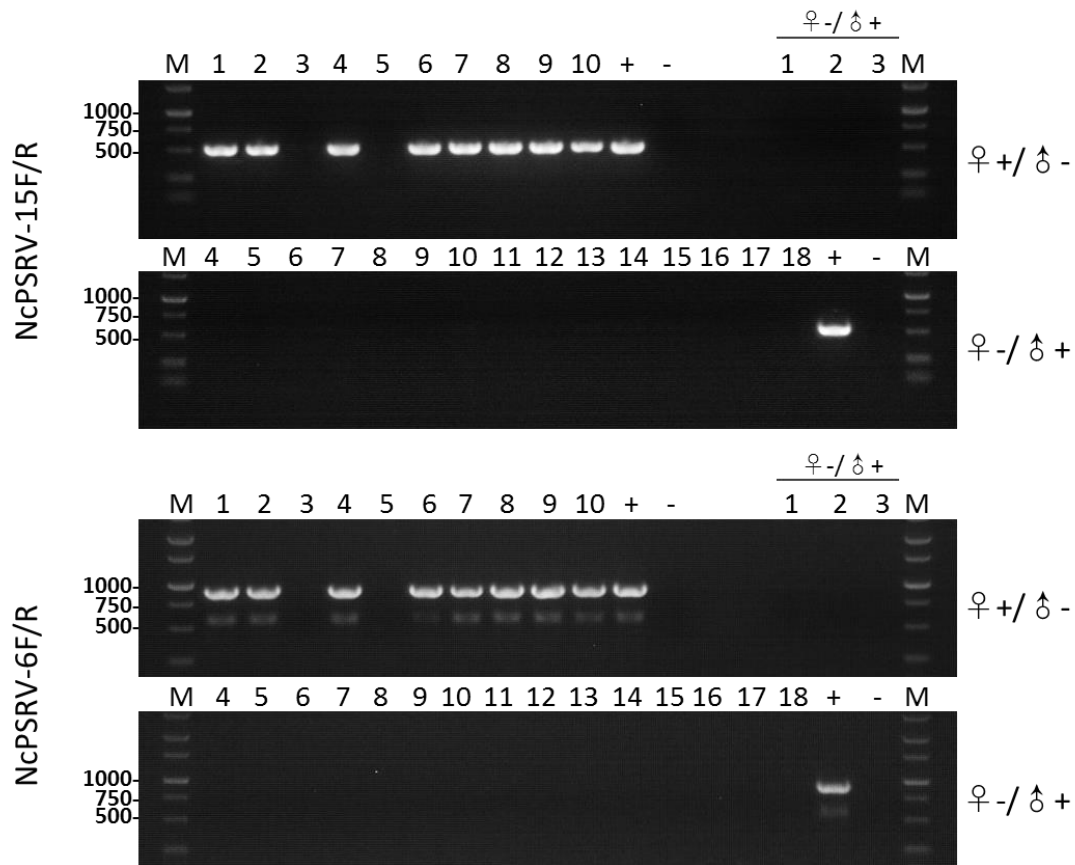

**B** *N. cincticeps* ♀ +/ ♂ +

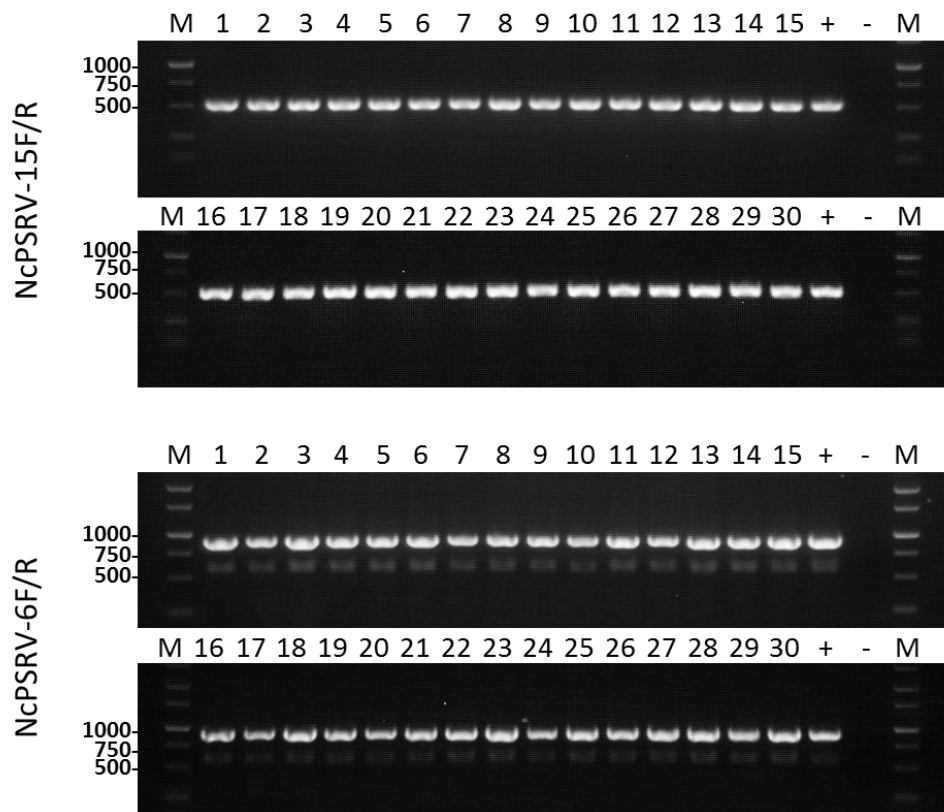

**C** *N. cincticeps* ♀-/ ♂-

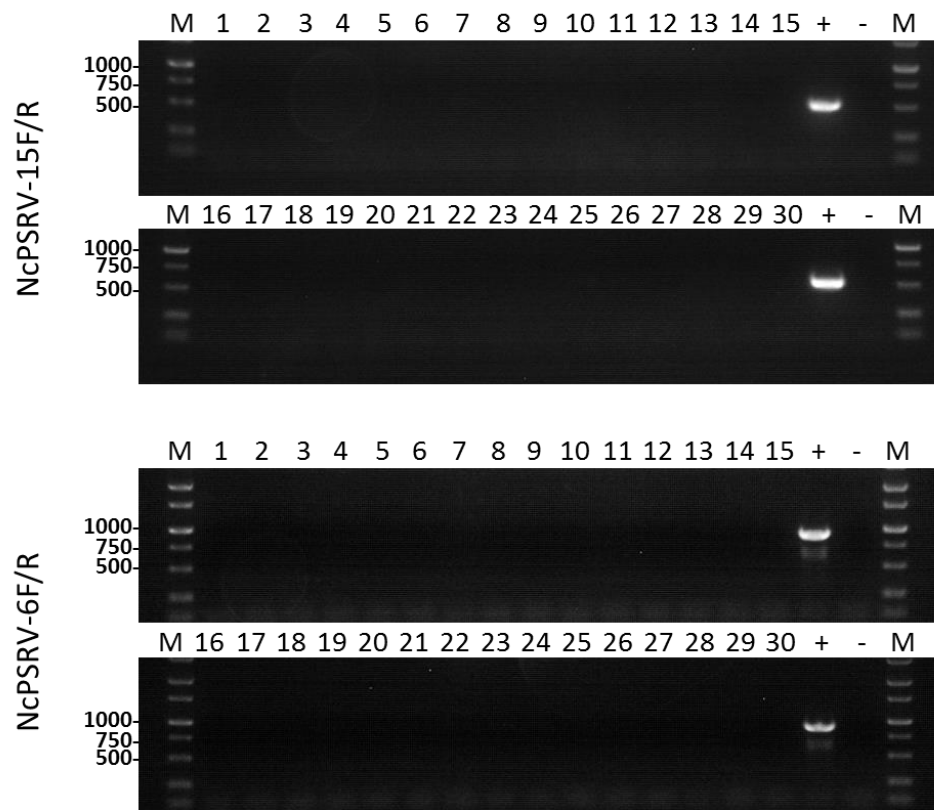

Supplement: Supplementary file 1 [file Data_Sheet_1.zip › Supplementary Material Presentation/Supplementary Figure S5.pdf]
